# *In vitro* upconverting/downshifting luminescent detection of tumor markers based on Eu^3+^-activated core–shell–shell lanthanide nanoprobes†
†Electronic supplementary information (ESI) available: Materials & methods, Fig. S1–S17 and Tables S1–S2. See DOI: 10.1039/c6sc01195k


**DOI:** 10.1039/c6sc01195k

**Published:** 2016-05-12

**Authors:** Yongsheng Liu, Shanyong Zhou, Zhu Zhuo, Renfu Li, Zhuo Chen, Maochun Hong, Xueyuan Chen

**Affiliations:** a Key Laboratory of Optoelectronic Materials Chemistry and Physics , Fujian Institute of Research on the Structure of Matter , Chinese Academy of Sciences , Fuzhou , Fujian 350002 , China . Email: xchen@fjirsm.ac.cn; b State Key Laboratory of Structural Chemistry , Fujian Institute of Research on the Structure of Matter , Chinese Academy of Sciences , Fuzhou , Fujian 350002 , China . Email: hmc@fjirsm.ac.cn

## Abstract

Trivalent europium (Eu^3+^) doped inorganic nanoparticles (NPs), emerging as a new class of red luminescent nanoprobes, have shown great promise in bioapplications as diverse as luminescent bioassays and disease theranostics owing to their superior optical properties such as long-lived downshifting luminescence (DSL) and upconverting luminescence (UCL). However, the exploration of Eu^3+^-doped NPs as red luminescent bioprobes particularly combined with DSL and UCL of Eu^3+^ hitherto remains untouched. Herein, we report a rational core–shell–shell (CSS) design strategy to construct Eu^3+^-activated NaGdF_4_:Yb/Tm@NaGdF_4_:Eu@NaEuF_4_ CSS NPs functionalized with efficient UCL and dissolution-enhanced DSL of Eu^3+^ for *in vitro* tumor marker detection and tumor-targeted imaging. By utilizing the CSS NPs as red luminescent nanoprobes, we demonstrate the successful UCL and DSL bioassays of a typical hepatic carcinoma biomarker, alpha-fetoprotein (AFP), in human serum samples. The UCL bioassay shows a limit of detection (LOD) of AFP down to 20 pg mL^–1^ (290 fM), which is the lowest among luminescent bioassays of AFP ever reported, and a 30-fold improvement relative to that of the commercial dissociation-enhanced lanthanide fluoroimmunoassay kit. Meanwhile the DSL bioassay, by employing the identical CSS NPs, can serve as a self-referential validation for the reliability and accuracy of the UCL bioassay for AFP detection. Furthermore, these CSS NPs can also function well in tumor-targeted UCL bioimaging, thereby revealing the great promise of the designed CSS NPs as red luminescent bioprobes in ultrasensitive *in vitro* detection of tumor markers in clinical diagnosis.

## Abstract 2

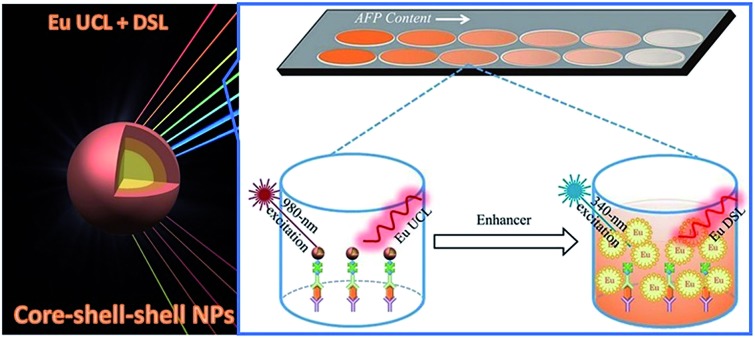
We demonstrate the successful use of Eu^3+^-activated core–shell–shell nanoprobes for *in vitro* upconverting/downshifting luminescent detection of a cancer biomarker of alpha-fetoprotein.

## Introduction

Primary hepatocellular carcinoma (HCC) is the sixth most common cancer and the second leading cause of cancer death worldwide.^1^ Alpha-fetoprotein (AFP) has been commonly used as a sensitive tumor marker for the early diagnosis and monitoring of primary HCC. The serum AFP levels for primary HCC patients often increase under conditions such as periods of rapid liver cancer cell growth, relapse and metastasis.^2^,^3^ For early stage diagnosis, an AFP level of >15 ng mL^–1^ is considered to be a reliable indicator for suspicion of primary HCC, while for monitoring the HCC relapse, the AFP level in the serum usually decreases to <20 ng mL^–1^ after the resection of HCC for the first 2 months, and a serial increase in the AFP level indicates the recurrence or metastasis of HCC.^4^ Therefore, ultrasensitive detection of AFP featuring a large linear range will have a significant impact on the theranostics of HCC. For this purpose, some tumor marker-detecting methods such as time-resolved fluoroimmunoassay and dissociation-enhanced lanthanide fluoroimmunoassay (DELFIA) have been well developed in the past decades by using lanthanide chelates as luminescent bioprobes.^2^,^5^–^7^ However, the use of these luminescent bioprobes somewhat suffers from disadvantages like poor photochemical stability and high cost-to-use.^8^

Compared to conventional molecular bioprobes, trivalent lanthanide (Ln^3+^)-doped inorganic nanoparticles (NPs) possess more fascinating chemical and optical properties such as long-lived multicolour emissions and photon upconversion ability, and thus are considered to be the most promising alternative to conventional probes for early cancer theranostics.^9^–^31^ In particular, trivalent europium (Eu^3+^) ion has been well established as the red-emitting element since the discovery of the bright red-emitting phosphor of Y_2_O_3_:Eu^3+^ at the beginning of the 20th century.^32^ Due to the wide energy gap between the luminescent and next low-lying energy levels (∼12 000 cm^–1^), the red emission of Eu^3+^ is usually typical of a long photoluminescence (PL) lifetime on the order of several milliseconds or longer, which makes the Eu^3+^-doped inorganic NPs highly suitable as sensitive time-resolved PL bioprobes for background-free biodetection (ESI, Fig. S1†).^33^,^34^ Nevertheless, for the vast majority of Eu^3+^-activated red bioprobes, the red emissions of Eu^3+^ are primarily produced *via* downshifting luminescence (DSL) processes upon ultraviolet (UV) excitation.^35^ By contrast, it is formidably difficult to achieve intense upconverting luminescence (UCL) of Eu^3+^ through the traditional approach by simple co-doping of Yb^3+^, due mainly to the large mismatch of the energy levels involved in the upconversion process (ESI, Fig. S2†). Recently, we have developed a general core–shell structured strategy to achieve intense UCL of Eu^3+^ in uniform NaGdF_4_:Yb/Tm@NaGdF_4_:Eu core–shell NPs by integrating the merits of core–shell nanostructures.^36^ Such unique UCL of Eu^3+^, possessing both advantages from long-lived PL lifetime and near-infrared (NIR) excitation, is substantially more resistant to the deleterious surface quenching than UCL from typical upconverting emitters like Er^3+^ and Tm^3+^ in diverse physiological environments, therefore showing greater promise in versatile bioapplications such as background-free UCL detection of tumor markers and tumor-targeting bioimaging.^37^,^38^

**Fig. 1:**
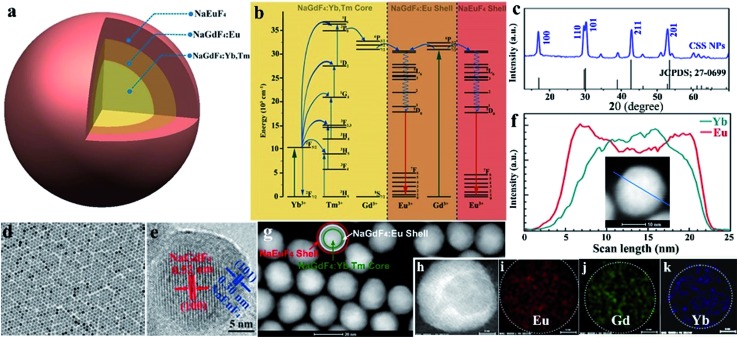
(a) Schematic design for the CSS nanoparticle comprised of a NaGdF_4_:Yb/Tm core and NaGdF_4_:Eu and NaEuF_4_ shells to produce intense UCL and DSL of Eu^3+^. (b) Proposed energy transfer mechanisms in the CSS NPs. Note that only partial energy levels of Eu^3+^, Yb^3+^, Tm^3+^ and Gd^3+^ are shown for clarity. (c) XRD pattern for the CSS NPs showing that all peaks can be well indexed in accordance with hexagonal-phase structure. (d) Low magnification and (e) high-resolution TEM images for the as-synthesized CSS NPs. (f) EELS line scan conducted with the HAADF-STEM image (inset) on a single CSS nanoparticle, indicating the formation of the CSS structured NPs. (g–k) HAADF-STEM images with elemental mapping results of the CSS NPs, demonstrating the contrast in density between the NaGdF_4_:Yb/Tm core, NaGdF_4_:Eu shell and NaEuF_4_ shell regions (marked by green, white and red circles, respectively) and the distributions of Eu^3+^, Gd^3+^ and Yb^3+^ within a single CSS nanoparticle.

Despite these important advances, red bioprobes based on Eu^3+^-doped inorganic NPs are mainly restricted to those NPs with single-mode luminescence, namely DSL, of Eu^3+^.^35^ Hitherto, the exploration of Eu^3+^-doped inorganic NPs as red luminescent bioprobes with combined UCL and DSL of Eu^3+^ has not been reported yet. The combined UCL and DSL strategy in single nanoparticles holds great promise in expanding the applicable range of Eu^3+^-doped red bioprobes for diverse bioapplications. Herein, we report a core–shell–shell (CSS) design strategy to fabricate Eu^3+^-activated red luminescent NaGdF_4_:Yb,Tm@NaGdF_4_:Eu@NaEuF_4_ CSS nanoprobes functionalized with both UCL and DSL of Eu^3+^ for *in vitro* detection of tumor markers and tumor-targeted imaging. Specifically, the NaGdF_4_:Yb/Tm core coupled with the NaGdF_4_:Eu shell is designed to achieve the unique red UCL of Eu^3+^ for the UCL bioassay of AFP in human serum samples (Fig. 1a and b), while the outermost NaEuF_4_ shell with highly concentrated Eu^3+^ ions is intentionally fabricated to produce intense dissolution-enhanced DSL of Eu^3+^ when mixing with the enhancer solution of 2-naphthoyltrifluoroacetone (β-NTA) for the DSL bioassay of AFP, which can serve as self-referential validation to evaluate the practicability of the UCL bioassay. Furthermore, we also demonstrate the successful use of these multifunctional CSS NPs as feasible red UCL bioprobes for targeted cancer cell imaging.

## Results and discussion

Monodisperse NaGdF_4_:Yb/Tm@NaGdF_4_:Eu@NaEuF_4_ CSS NPs were fabricated by employing a layer-by-layer epitaxial growth strategy as previously reported,^39^,^40^ which involved the growth of 13 ± 1.8 nm NaGdF_4_:Yb/Tm (20/1 mol%) core NPs followed by the successive deposition of two epitaxial shells of NaGdF_4_:Eu (15 mol%) and NaEuF_4_ (ESI, Fig. S3†). The as-synthesized CSS NPs were determined to be in hexagonal phase by powder X-ray diffraction analysis (Fig. 1c). The representative transmission electron microscopy (TEM) image for the CSS NPs exhibits uniform size distribution and nearly spherical shape with an average size of 23 ± 2.9 nm (Fig. 1d). The corresponding high-resolution TEM image of an individual nanoparticle displays clear lattice fringes with an observed *d*-spacing of 0.52 and 0.30 nm (Fig. 1e), which agrees well with the lattice spacing of the (100) and (101) planes for the hexagonal phase NaGdF_4_ and NaEuF_4_ crystals, respectively, thereby to some extent verifying the formation of the CSS structure. This finding was validated by electron energy loss spectroscopy (EELS) conducted with a high angle annular dark-field scanning TEM (HAADF-STEM) image that demonstrates the content variation of Eu and Yb across a randomly selected nanoparticle, where the higher Eu content at the peripheral region and higher Yb content in the interior region are basically consistent with the designed compositions for the CSS structure (Fig. 1f). Accordingly, the HAADF-STEM image for the CSS NPs shows a slightly discernible contrast of the CSS structure (Fig. 1g) and different elemental distributions for the NaGdF_4_:Yb/Tm core, NaGdF_4_:Eu and NaEuF_4_ shell regions within a single CSS nanoparticle (Fig. 1h–k).

**Fig. 2:**
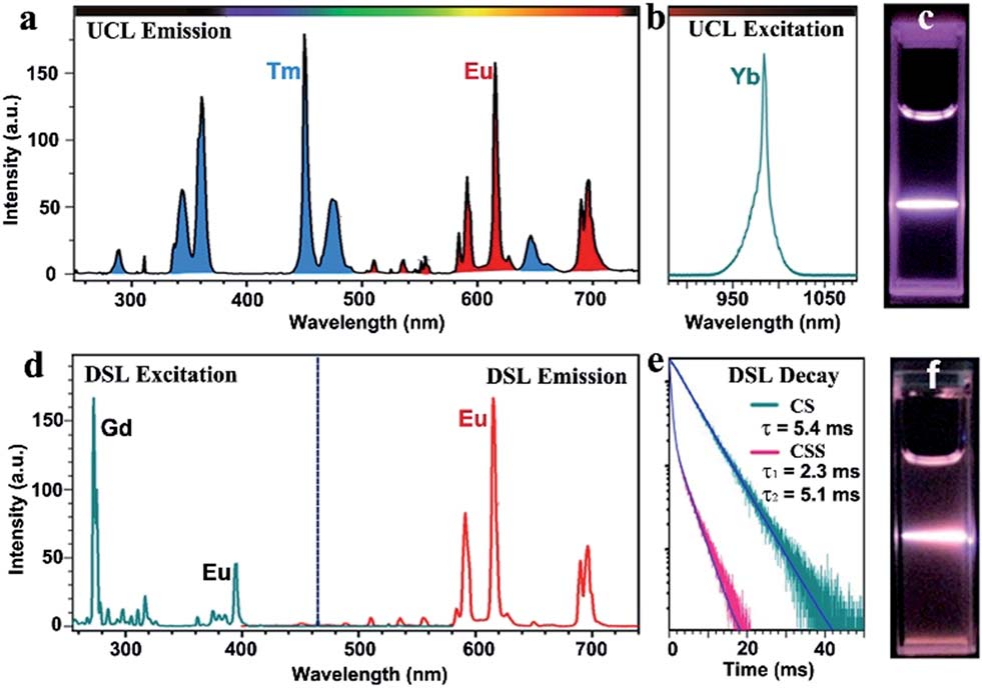
UCL (a) emission and (b) excitation spectra showing the Yb^3+^ sensitized UCL of Eu^3+^ (red) and Tm^3+^ (blue) in the as-synthesized CSS NPs, and (c) the corresponding UCL photograph for the as-synthesized CSS NPs dispersed in cyclohexane under NIR laser irradiation at 980 nm with a power density of 10 W cm^–2^. (d) DSL excitation (right) and emission spectra showing the host Gd^3+^-sensitized DSL of Eu^3+^, and (e) DSL decays from ^5^D_0_ of Eu^3+^ in the CS and CSS NPs by monitoring the Eu^3+^ emission at 615 nm upon UV excitation at 273 nm. (f) The corresponding DSL photograph for the as-synthesized CSS NPs dispersed in cyclohexane under laser irradiation at 273 nm with a power density of 10 mW cm^–2^.

To demonstrate the feasibility of our design strategy, we investigated the PL properties of the as-synthesized CSS NPs. Upon 980 nm NIR laser irradiation, characteristic UCL peaks attributed to the intra-4f transitions of Eu^3+^ (red) and Tm^3+^ (blue) sensitized by Yb^3+^ (Fig. 2a and b), which produces a pink color output (Fig. 2c) that is visible to the naked eye with superior photostability (ESI, Fig. S4†), can be readily achieved for the as-synthesized CSS NPs. Note that the UCL intensity for the CSS NPs is about 2.6 and 1.7 times stronger than those of NaGdF_4_:Yb/Tm@NaGdF_4_:Eu and NaGdF_4_:Yb/Tm@NaEuF_4_ core–shell counterparts (ESI, Fig. S5†). The UCL absolute quantum yield (QY), defined as the ratio of the number of emitted photons to the number of absorbed photons, was determined to be 3.3% upon NIR excitation using a 980 nm laser with a power density of 100 W cm^–2^. By contrast, upon indirect UV excitation of Gd^3+^ at 273 nm, a series of typical DSL peaks were observed with a dominant red emission band centered at 615 nm (Fig. 2d, right), which can be exclusively assigned to the ^5^D_0_ → ^7^F_2_ transition of Eu^3+^, indicating the existence of energy transfer from Gd^3+^ to Eu^3+^ (Fig. 2d, left).^41^,^42^ These emission peaks may arise from the overlapping of optical emissions from all Eu^3+^ ions residing in both the NaGdF_4_:Eu and NaEuF_4_ shells, considering the specific CSS structure we intentionally designed. To validate this, we measured the DSL decays of Eu^3+^ in NaEuF_4_ core-only (ESI, Fig. S6†), NaGdF_4_:Yb/Tm@NaGdF_4_:Eu core–shell and their corresponding CSS NPs. As compared in Fig. 2e, the DSL decay from Eu^3+^ in the core–shell NPs exhibited a single-exponential nature, indicative of the nearly homogeneous crystal-field environment around Eu^3+^ in the core–shell NPs. The DSL lifetime of Eu^3+^ in the core–shell NPs was determined to be 5.4 ms by a single-exponential fit to the decay. In stark contrast, a bi-exponential decay behavior of Eu^3+^ that is composed of a fast component in the initial stage and a much slower component in the tail was observed for the CSS NPs, largely due to the different crystal-field surroundings experienced by Eu^3+^ ions in the NaGdF_4_ and NaEuF_4_ shells. By fitting with a double exponential function, the DSL lifetimes were estimated to be 2.3 and 5.1 ms for the fast and slow decays of Eu^3+^ in the CSS NPs, respectively, which agree basically with the NaEuF_4_ core-only (2.2 ms) and their core–shell counterparts (5.4 ms). Similar results were observed for the UCL decays of Eu^3+^ in the CS and CSS NPs (ESI, Fig. S7†). As such, we can conclude that the DSL observed in Fig. 2f is really attributed to Eu^3+^ ions located in both the NaGdF_4_:Eu and NaEuF_4_ shells, which further confirms the formation of the CSS structure.

To render the hydrophobic as-synthesized CSS NPs hydrophilic and biocompatible, we removed the original capping ligand (oleic acid) from their surfaces *via* a well-developed acid-washing procedure.^43^ By doing so, the ligand-free CSS NPs with positively charged Eu^3+^ exposed on their surfaces were obtained, as evidenced by the positive *ζ*-potential of +46.7 mV when dispersed in water at pH 6.9 (ESI, Fig. S8†). These ligand-free CSS NPs exhibited much better water solubility as compared to their NaGdF_4_:Yb/Tm@NaGdF_4_:Eu core–shell counterparts and could be steadily dispersed in distilled water for several months. By virtue of dynamic light scattering analysis, the average hydrodynamic diameter for these ligand-free CSS NPs was determined to be 22 ± 1.9 nm (ESI, Fig. S9†), which is basically consistent with estimation from the TEM images.

Thanks to the bare Eu^3+^ ions on the surface, these ligand-free NPs can be directly conjugated with diverse biocompatible molecules like biotin with electronegative groups through electrostatic interaction.^43^ The successful conjugation of biotin to the nanoparticle surface was corroborated by the *ζ*-potentials, Fourier transform infrared spectra as well as the thermogravimetric analysis for the ligand-free NPs before and after surface modification (ESI, Fig. S8–S11†). By using an avidin/4′-hydroxyazobenzene-2-carboxylic acid reagent, the amount of biotin attached to the NPs was determined to be 1.2 μg mg^–1^, and the number of biotin molecules per NP was calculated to be 8.1 (ESI, Fig. S12†). As a result, the biotinylated CSS NPs can indirectly capture the biotinylated biomolecules upon addition of avidin through the strong biotin–avidin–biotin interaction. More importantly, these biotinylated CSS NPs were found to preserve the intense UCL of their parent NPs due to the effective protection of the outermost NaEuF_4_ shell against surface quenching from the attached biotin and solvent molecules (ESI, Fig. S13†), indicating the excellent UCL performance of the Eu^3+^-activated red luminescent bioprobes we developed.

**Fig. 3:**
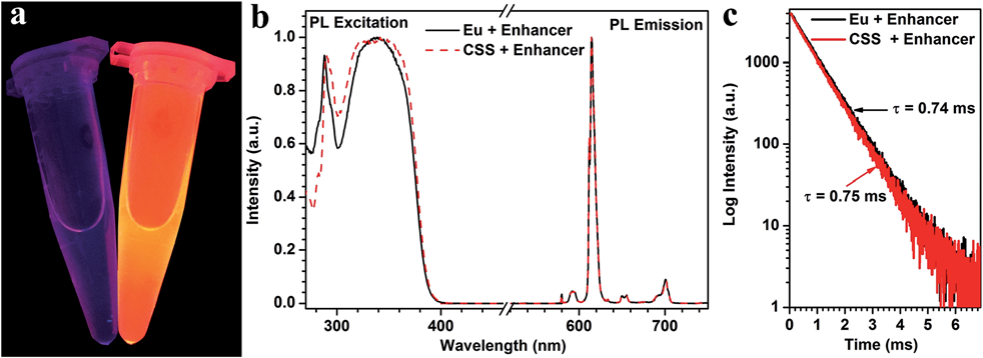
(a) DSL photographs of the biotinylated CSS NPs dispersed in water (left) and the enhancer solution (right) under UV-lamp illumination at 340 nm. (b) Normalized PL excitation spectra and emission spectra for the biotinylated CSS NPs dispersed in enhancer solution and the β-NTA–Eu^3+^–TOPO complex, respectively. Note that the PL excitation spectra were recorded by monitoring the Eu^3+^ emission at 615 nm, while the PL emission spectra were recorded under UV excitation at 340 nm. (c) Corresponding DSL decays from ^5^D_0_ of Eu^3+^ by monitoring the red emission at 615 nm.

To examine whether these biotinylated CSS NPs can be used as feasible red DSL bioprobes, we subsequently investigated their dissociation-enhanced PL behaviors after dispersing them in an enhancer solution (pH 2.3) containing Triton X-100, tri-*n*-octylphosphine oxide (TOPO) and β-NTA. As shown in Fig. 3a, these aforementioned biotinylated CSS NPs (20 μg mL^–1^) display bright red DSL upon 340 nm lamp illumination when mixing with the enhancer solution, which differs markedly from their original counterparts dispersed in water, where the red DSL is nearly invisible to the naked eye. By utilizing a microplate reader, the red DSL signal of Eu^3+^ for the biotinylated CSS NPs in the enhancer solution was determined to be ∼10^6^ times stronger than that dispersed in water (Table S1†). As a result, the DSL absolute QY significantly increased from 0.1% to 29.5% before and after the addition of the enhancer solution. Such dissociation-enhanced DSL of Eu^3+^ is owing to the formation of numerous highly luminescent β-NTA–Eu^3+^–TOPO complexes when mixing the biotinylated CSS NPs with the enhancer solution,^44^ as evidenced by their almost identical PL excitation, emission spectra and lifetime with those of the solution of the β-NTA–Eu^3+^–TOPO complex synthesized by direct mixing of europium acetate with the enhancer solution (Fig. 3b and c). Due to the high molar extinction coefficient of β-NTA (5.82 × 10^4^ cm^–1^ M^–1^) at ∼340 nm,^45^ the newly formed β-NTA–Eu^3+^–TOPO complex can act as an effective light-harvesting antenna for UV excitation light and transfer energy to the coordinated Eu^3+^, thereby giving rise to significantly amplified red DSL of Eu^3+^. Besides, it was found that the red DSL signal of Eu^3+^ increased linearly with the CSS NP concentration in the large range of 0–20 μg mL^–1^ (ESI, Fig. S14†) and can reach the maximum within 4 minutes even with a concentration as high as 20 μg mL^–1^ (ESI, Fig. S15†). These results reveal the superb dissolution-enhanced DSL performance for the biotinylated CSS NPs, which thereby enable the sensitive detection of tumor markers in human serum samples by using the DSL bioassay.^34^

**Fig. 4:**
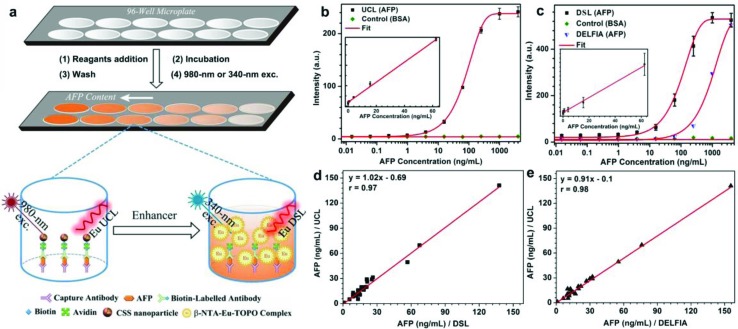
(a) Schematic illustration of the proposed UCL and DSL bioassays of AFP based on the UCL and dissociation-enhanced DSL of Eu^3+^ in the biotinylated CSS NPs. (b) Calibration curves for AFP detection *via* the UCL bioassay based on the biotinylated CSS NPs. (c) Calibration curves for AFP detection *via* the DSL bioassay based on the biotinylated CSS NPs and commercial DELFIA kit based on the Eu^3+^–DTTA complex, respectively. The control experiments in (b) and (c) were conducted by using BSA instead of the AFP antigen as the analyte under otherwise identical conditions. By contrast, the UCL and DSL signals were hardly detectable due to the lack of specific binding between BSA and anti-AFP antibody. The insets of (b) and (c) show the corresponding linear range of the calibration curve for AFP detection *via* UCL and DSL bioassays. (d and e) Correlations between the UCL bioassay, DSL bioassay and commercial DELFIA kit for the AFP detection in 20 human serum samples, where the ordinate and abscissa denote the AFP level determined in UCL bioassay, DSL bioassay and DELFIA kit, respectively.

As a proof-of-concept experiment, we employed the biotinylated CSS NPs as UCL and DSL red nanoprobes to detect the tumor marker of AFP in human serum samples *via* a sandwich-type bioassay. The principles for the sandwich-type UCL and DSL are schematically illustrated in Fig. 4a and S16 (ESI†). In a typical procedure, the capture anti-AFP antibody was first immobilized on a 96-well microplate *via* incubation, and then conjugated with the analyte solution of the AFP antigen in view of their specific recognition and high binding affinity. Subsequently, the solution containing the biotinylated detection anti-AFP antibody that was itself bound to the biotinylated nanoprobe *via* the linker of avidin was added in order to label the analyte. As a result, the concentration of AFP can be quantified by measuring the UCL signals of Eu^3+^ for the nanoprobes bound to a 96-well microplate upon 980 nm excitation on our customized UCL biodetection system integrated with a microplate reader (Synergy 4, BioTek). After the UCL bioassay, AFP was further quantified by measuring the dissolution-enhanced DSL signals of Eu^3+^ upon addition of the enhancer solution *via* the DSL bioassay that has recently been developed as one of the most sensitive luminescent bioassay techniques.^34^ As shown in Fig. 4b and c, both the UCL and DSL signals of Eu^3+^ in the calibration curves for AFP detection were found to gradually increase with the amount of AFP antigen bound to the microplate wells and tend to saturate when the concentration exceeds 1000 ng mL^–1^. In particular, the calibration curves for the UCL bioassay exhibited excellent linear dependence in a large concentration range spanning about 4 orders of magnitude (0.01–60 ng mL^–1^). For comparison, we employed the commercially available DELFIA kit for the AFP assay based on the molecular probe of the Eu^3+^–DTTA complex (ESI, Fig. S16†). In comparison with DELFIA (Fig. 4c, blue), the inflection point in the calibration curve for either the UCL or DSL bioassay appeared at the much lower concentration, indicating a lower limit of detection (LOD) of AFP. The LOD, defined as the concentration that corresponds to three times the standard deviation above the signal measured in the control experiments (Fig. 4b and c), was determined to be approximately 20 pg mL^–1^ (290 fM) for the UCL bioassay and 60 pg mL^–1^ (870 fM) for the DSL bioassay, respectively. To the best of our knowledge, the LOD of 290 fM is the lowest among luminescent bioassays of AFP ever reported^2^,^46^,^47^ and is 30 times lower than that of the commercial DELFIA kit (600 pg mL^–1^). The UCL bioassay makes use of the 980 nm NIR diode laser as the excitation source, where the biological cells and tissues have minimal absorption and thus auto-fluorescence derived from the UV excitation can be thoroughly avoided in the detection, thereby resulting in much higher detection sensitivity as compared to the DELFIA kit or DSL bioassay. Such a low LOD coupled with the wide linear detection range of AFP aforementioned for the UCL bioassay is highly desirable to fulfil the critical requirement of tracing the serum AFP levels in the intervals for early diagnosis of HCC and monitoring HCC relapse after hepatectomy. Furthermore, it is worth emphasizing that, despite achieving a slightly higher LOD, the DSL bioassay by employing the identical CSS NPs may act as a self-referential validation for evaluating the applicability of the UCL bioassay.

To verify the applicability of our red nanoprobes in practical bioassays, we conducted *in vitro* detection of the AFP levels in 20 human serum samples from normal humans and cancer patients *via* the UCL bioassay. The AFP levels determined from the UCL bioassay were then compared with those independently measured *via* the DSL bioassay and commercial DELFIA kit (Table S2†). As compared in Fig. 4d and e, the AFP levels from the UCL bioassay are very consistent with those from the DELFIA kit and DSL bioassay. The correlation coefficient between the UCL bioassay and the DSL bioassay (or DELFIA) were determined to be 0.97 (or 0.98), indicating that the UCL bioassay results are as reliable as those of the DSL bioassay and commercial DELFIA kit. Furthermore, we evaluated the analytical accuracy and precision of the UCL bioassay through the determination of the AFP level, coefficient of variation (CV), and the recovery of one human serum sample upon addition of human AFP standard solutions with different concentrations. The CVs of all assays are below 5% and the analytical recoveries are in the range of 102–109% (Table 1), both of which are within the acceptance criteria (CVs ≤ 15%; recoveries in the range of 90–110%) set for the bioanalytical method validation,^48^ thereby verifying the high reliability and practicability of the red luminescent CSS nanoprobes we developed for AFP detection.

**Table 1:** Assay precision and analytical recovery of AFP added to serum samples

	Added (ng mL^–1^)	Found (ng mL^–1^)	CV^*a*^ (%) (*n* = 4)	Recovery (%)
UCL bioassay	0	5.1	2.36	—
	0.5	5.6	3.28	108.1
	10.7	16.2	4.42	103.7
	56.0	62.4	4.40	102.3

^*a*^Intra-assay using four different wells in a plate.

**Fig. 5:**
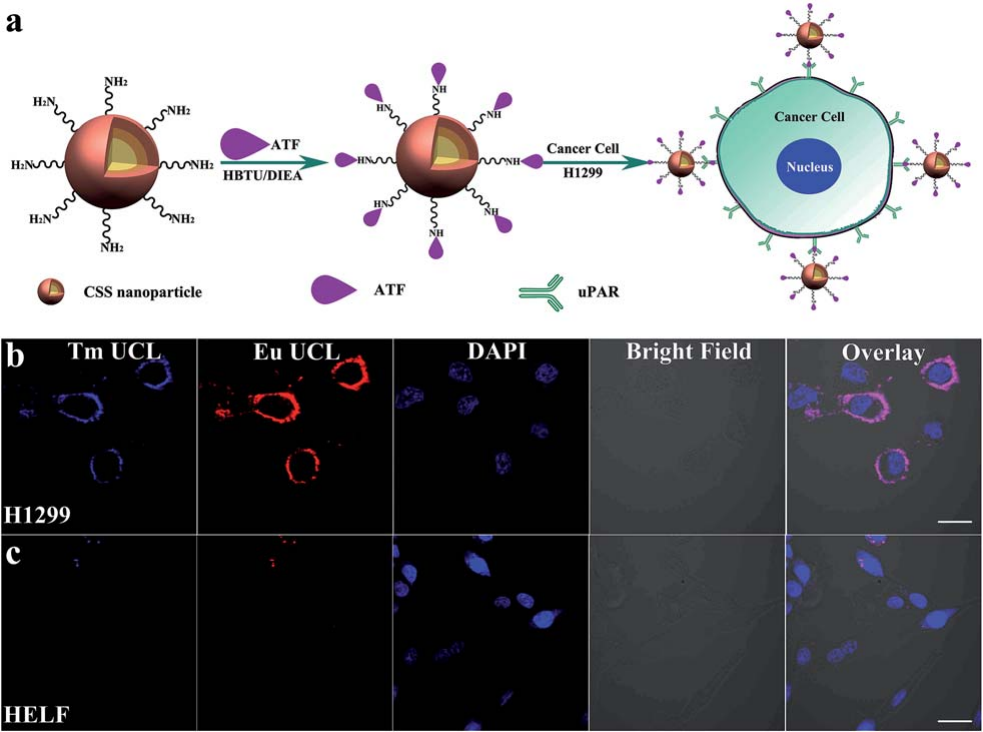
(a) Schematic illustration showing the bioconjugation of amine-functionalized CSS NPs with ATF and specific recognition of ATF-coupled CSS NPs to H1299 cancer cells with uPAR highly-expressed on the membrane. CLSM UCL (*λ*_ex_ = 980 nm) images of H1299 (b) and HELF (c) cells after incubation with ATF-coupled CSS NPs (200 μg mL^–1^) for 2 h at 37 °C. Blue Tm and red Eu UCL signals for the CLSM images were collected in the spectroscopy region of 450–490 nm and 590–630 nm, respectively, and DAPI (4′,6-diamidino-2-phenylindole) blue (*λ*_ex_ = 405 nm) and bright-field images were used to outline the nuclear regions and cell positions, respectively (scale bar = 30 μm).

To demonstrate the multifunctionality of the red luminescent CSS nanoprobes, we also employed the CSS NPs as tracers in tumor-targeted bioimaging by utilizing the intense UCL of Eu^3+^ after coupling them with the amino-terminal fragment (ATF) of the urokinase plasminogen activator (an important marker of tumor biology and metastasis) (ESI†).^49^ Thanks to the high binding affinity between ATF and urokinase plasminogen activator receptor (uPAR),^50^ the ATF-coupled CSS NPs can be specifically targeted to the membrane of human lung cancer cells (H1299) with uPAR high expression after co-incubation in a culture medium at 37 °C for 2 h (Fig. 5a).^51^ As a result, intense red UCL from Eu^3+^ can be clearly visualized in the membranes of H1299 cells upon 980 nm laser irradiation using CLSM (Fig. 5b). For comparison, we conducted the control experiments under otherwise identical conditions by using human embryo lung fibroblast (HELF) cells with low uPAR expression on their membrane, in which the red UCL signal of Eu^3+^ was hardly observable due to the lack of specific recognition between the ATF-coupled CSS NPs and HELF cells (Fig. 5c). These results clearly demonstrate that ATF-coupled CSS NPs can be successfully used as red bioprobes for targeted imaging of cancer cells based on the UCL of Eu^3+^, which had not been realized before. The phototoxicity and dark and real-time cytotoxicity of ATF-coupled CSS NPs were further assessed on HELF cells by means of the cell-counting-kit (CCK-8) and electric cell-substrate impedance sensing assays (ESI, Fig. S17†). The cell viability was determined to be larger than 90% even at a NP concentration as high as 500 μg mL^–1^. Accordingly, the impedance of HELF cells did not change significantly with increased time, indicating that no apparent cell death was induced after co-incubation of the ATF-coupled CSS NPs with HELF cells. Such a low toxicity thus infers that the multifunctional nano-bioprobes we developed are biocompatible and virtually nontoxic to live cells.

## Conclusions

In summary, we have developed a universal strategy to fabricate Eu^3+^-activated red luminescent NaGdF_4_:Yb,Tm@NaGdF_4_:Eu@NaEuF_4_ CSS nanoprobes for *in vitro* detection of tumor markers and tumor-targeted imaging. The NaGdF_4_:Yb/Tm core coupled with the NaGdF_4_:Eu inner shell was designed to achieve the unique red UCL of Eu^3+^ upon 980 nm NIR excitation for UCL bioassay of AFP in human serum samples, and the outermost NaEuF_4_ shell with highly concentrated Eu^3+^ ions was intentionally fabricated to yield significantly amplified DSL of Eu^3+^ when mixing with the enhancer solution for the DSL bioassay. The UCL bioassay exhibited a LOD down to 20 pg mL^–1^, which was about 3 and 30 times lower than those determined from the self-referential DSL bioassay and commercial DELFIA kit, respectively. More importantly, we have successfully employed the red luminescent CSS nanoprobes for the ultrasensitive detection of AFP in 20 clinical serum samples of normal humans and HCC patients. It was found that the UCL assay results were quite consistent with those measured independently by the DSL bioassay and DELFIA kit, showing the assay's reliability with correlation coefficients of 0.97 and 0.98, respectively. Meanwhile, we have also demonstrated the successful use of these Eu^3+^-activated CSS nanoprobes in tumor-targeted imaging based on the UCL of Eu^3+^. These findings demonstrate the great potential of such a red luminescent nano-bioprobe in practical *in vitro* detection of tumor markers,^52^ particularly, for monitoring HCC relapse of patients after hepatectomy, which may pave the way for the exploration of inorganic lanthanide NPs in versatile bioapplications and eventually impact the community or family medical practice.

## Supplementary Material

Supplementary information

## References

[cit1] Stewart B. W. and Wild C. P., World Cancer Report 2014, World Health Organization, 2014.

[cit2] Wu B. Y., Wang H. F., Chen J. T., Yan X. P. (2011). J. Am. Chem. Soc..

[cit3] Qin L. X., Tang Z. Y. (2004). J. Cancer Res. Clin. Oncol..

[cit4] Terentiev A. A., Moldogazieva N. T. (2013). Tumor Biol..

[cit5] Charbonniere L. J., Hildebrandt N., Ziessel R. F., Loehmannsroeben H. G. (2006). J. Am. Chem. Soc..

[cit6] Matsumoto K., Yuan J. G., Wang G. L., Kimura H. (1999). Anal. Biochem..

[cit7] Bunzli J. C. G. (2010). Chem. Rev..

[cit8] Chen Z., Zheng W., Huang P., Tu D. T., Zhou S. Y., Huang M. D., Chen X. Y. (2015). Nanoscale.

[cit9] Dong H., Sun L.-D., Yan C.-H. (2015). Chem. Soc. Rev..

[cit10] Gorris H. H., Wolfbeis O. S. (2013). Angew. Chem., Int. Ed..

[cit11] Deng R., Qin F., Chen R., Huang W., Hong M., Liu X. (2015). Nat. Nanotechnol..

[cit12] Gai S., Li C., Yang P., Lin J. (2014). Chem. Rev..

[cit13] Zhou J., Liu Q., Feng W., Sun Y., Li F. (2015). Chem. Rev..

[cit14] Gorris H. H., Ali R., Saleh S. M., Wolfbeis O. S. (2011). Adv. Mater..

[cit15] Haase M., Schafer H. (2011). Angew. Chem., Int. Ed..

[cit16] Sun L. D., Wang Y. F., Yan C. H. (2014). Acc. Chem. Res..

[cit17] Zhang F., Shi Q. H., Zhang Y. C., Shi Y. F., Ding K. L., Zhao D. Y., Stucky G. D. (2011). Adv. Mater..

[cit18] Tsang M.-K., Bai G., Hao J. (2015). Chem. Soc. Rev..

[cit19] Li P., Peng Q., Li Y. D. (2009). Adv. Mater..

[cit20] Yang D., Ma P. a., Hou Z., Cheng Z., Li C., Lin J. (2015). Chem. Soc. Rev..

[cit21] Lu Y., Zhao J., Zhang R., Liu Y., Liu D., Goldys E. M., Yang X., Xi P., Sunna A., Lu J., Shi Y., Leif R. C., Huo Y., Shen J., Piper J. A., Robinson J. P., Jin D. (2013). Nat. Photonics.

[cit22] Zhou B., Shi B., Jin D., Liu X. (2015). Nat. Nanotechnol..

[cit23] Dai Y. L., Xiao H. H., Liu J. H., Yuan Q. H., Ma P. A., Yang D. M., Li C. X., Cheng Z. Y., Hou Z. Y., Yang P. P., Lin J. (2013). J. Am. Chem. Soc..

[cit24] Gai S. L., Yang P. P., Li C. X., Wang W. X., Dai Y. L., Niu N., Lin J. (2010). Adv. Funct. Mater..

[cit25] Liu X. W., Deng R. R., Zhang Y. H., Wang Y., Chang H. J., Huang L., Liu X. G. (2015). Chem. Soc. Rev..

[cit26] Teng X., Zhu Y. H., Wei W., Wang S. C., Huang J. F., Naccache R., Hu W. B., Tok A. I. Y., Han Y., Zhang Q. C., Fan Q. L., Huang W., Capobianco J. A., Huang L. (2012). J. Am. Chem. Soc..

[cit27] Park Y. I., Lee K. T., Suh Y. D., Hyeon T. (2015). Chem. Soc. Rev..

[cit28] Idris N. M., Gnanasammandhan M. K., Zhang J., Ho P. C., Mahendran R., Zhang Y. (2012). Nat. Med..

[cit29] Xia L., Kong X. G., Liu X. M., Tu L. P., Zhang Y. L., Chang Y. L., Liu K., Shen D. Z., Zhao H. Y., Zhang H. (2014). Biomaterials.

[cit30] Schafer H., Ptacek P., Zerzouf O., Haase M. (2008). Adv. Funct. Mater..

[cit31] Wu X., Zhu W. (2015). Chem. Soc. Rev..

[cit32] Eliseeva S. V., Bunzli J. C. G. (2010). Chem. Soc. Rev..

[cit33] Liu Y. S., Tu D. T., Zhu H. M., Chen X. Y. (2013). Chem. Soc. Rev..

[cit34] Zhou S. Y., Zheng W., Chen Z., Tu D. T., Liu Y. S., Ma E., Li R. F., Zhu H. M., Huang M. D., Chen X. Y. (2014). Angew. Chem., Int. Ed..

[cit35] Shen J., Sun L. D., Zhu J. D., Wei L. H., Sun H. F., Yan C. H. (2010). Adv. Funct. Mater..

[cit36] Liu Y. S., Tu D. T., Zhu H. M., Li R. F., Luo W. Q., Chen X. Y. (2010). Adv. Mater..

[cit37] Wang F., Deng R. R., Wang J., Wang Q. X., Han Y., Zhu H. M., Chen X. Y., Liu X. G. (2011). Nat. Mater..

[cit38] Li Z., Liang T., Lv S. W., Zhuang Q. G., Liu Z. H. (2015). J. Am. Chem. Soc..

[cit39] Johnson N. J. J., Korinek A., Dong C. H., van Veggel F. C. J. M. (2012). J. Am. Chem. Soc..

[cit40] Zhang F., Che R. C., Li X. M., Yao C., Yang J. P., Shen D. K., Hu P., Li W., Zhao D. Y. (2012). Nano Lett..

[cit41] Wegh R. T., Donker H., Oskam K. D., Meijerink A. (1999). Science.

[cit42] Ptacek P., Schafer H., Kompe K., Haase M. (2007). Adv. Funct. Mater..

[cit43] Bogdan N., Vetrone F., Ozin G. A., Capobianco J. A. (2011). Nano Lett..

[cit44] Xu J., Zhou S., Tu D., Zheng W., Huang P., Li R., Chen Z., Huang M., Chen X. (2016). Chem. Sci..

[cit45] Gschneidner K. A., Bünzli J.-C. G. and Pecharsky V. K., Handbook on the Physics and Chemistry of Rare Earths, Elsevier Press, North-holland, 2007, vol. 37.

[cit46] Chen H. Q., Guan Y. Y., Wang S. Z., Ji Y., Gong M. Q., Wang L. (2014). Langmuir.

[cit47] Yuan J., Wang G., Majima K., Matsumoto K. (2001). Anal. Chem..

[cit48] Shah V. P., Midha K. K., Findlay J. W. A., Hill H. M., Hulse J. D., McGilveray I. J., McKay G., Miller K. J., Patnaik R. N., Powell M. L., Tonelli A., Viswanathan C. T., Yacobi A. (2000). Pharm. Res..

[cit49] Zheng W., Zhou S. Y., Chen Z., Hu P., Liu Y. S., Tu D. T., Zhu H. M., Li R. F., Huang M. D., Chen X. Y. (2013). Angew. Chem., Int. Ed..

[cit50] Goodson R. J., Doyle M. V., Kaufman S. E., Rosenberg S. (1994). Proc. Natl. Acad. Sci. U. S. A..

[cit51] Chen Z., Lin L., Huai Q., Huang M. D. (2009). Comb. Chem. High Throughput Screening.

[cit52] Zheng W., Huang P., Tu D. T., Ma E., Zhu H. M., Chen X. Y. (2015). Chem. Soc. Rev..

