# Supplementary material for: In vitro upconverting/downshifting luminescent detection of tumor markers based on Eu3+-activated core–shell–shell lanthanide nanoprobes
Source: Chem Sci. 2016 May 12;7(8):5013–9. doi: 10.1039/c6sc01195k (PMC6018526; doi:10.1039/c6sc01195k)
Supplement: Supplementary file 1 [file SC-007-C6SC01195K-s001.pdf]

Electronic Supplementary Material (ESI) for Chemical Science.
This journal is © The Royal Society of Chemistry 2016

Electronic Supplementary Information (ESI) for Chemical Science

In-Vitro Upconverting/Downshifting Luminescent Detection of Tumor Markers Based on Eu^{3+}-Activated Core-Shell-Shell Lanthanide Nanoprobes

Yongsheng Liu,[a,b] Shanyong Zhou,[a] Zhu Zhuo,[b] Renfu Li,[a] Zhuo Chen,[b] Maochun Hong,*[b] and Xueyuan Chen*[a,b]

[a.]Key Laboratory of Optoelectronic Materials Chemistry and Physics, Fujian Institute of Research on the Structure of Matter, Chinese Academy of Sciences, Fuzhou, Fujian 350002, China

[b.]State Key Laboratory of Structural Chemistry, Fujian Institute of Research on the Structure of Matter, Chinese Academy of Sciences, Fuzhou, Fujian 350002, China

*To whom correspondence should be addressed, e-mail: xchen@fjirsm.ac.cn and hmc@fjirsm.ac.cn

I. Supplementary Materials & Methods

Chemicals and Materials: $Ln(CH_3COO)_3 \cdot 4H_2O$ (Ln=Gd, Yb, Eu and Tm), 1-octadecene (ODE), oleic acid (OA), 2-naphthoyltrifluoroacetone (β-NTA) and tri-n-octylphosphine oxide (TOPO), 2-aminoethyl dihydrogen phosphate (AEP), 4′-hydroxyazobenzene-2-carboxylic acid, o-Benzotriazole-N,N,N’,N’-tetramethyl-uronium-hexafluoro-phosphate, N,N-diisopropylethylamine, 4',6-diamidino-2-phenylindole (DAPI), biotin, avidin, Triton X-100, TWEEN 20 and bovine serum albumin (BSA) were purchased from Sigma-Aldrich (China). NaOH, NH_4F, N,N-dimethylformamide (DMF) and ethanol were purchased from Sinopharm Chemical Reagent Co., China. The standard solutions of alphafetoprotein (AFP), mouse anti-AFP monoclonal antibody, and biotinylated mouse anti-AFP monoclonal antibody were purchased from Shanghai Linc-Bio Science Co. Dissociation-Enhanced Lanthanide Fluoroimmunoassay (DELFIA) kit for AFP was purchased from Daan Gene Co., Ltd. of Sun Yat-Sen University, China (http://www.daruiantibody.com). Human serum samples were kindly provided by Fujian Provincial Cancer Hospital, Fuzhou, China. The 96-well Nunc Immobilizer Amino plate was purchased from Thermo Fisher Scientific Inc., which has a high affinity for the coupling of peptide and protein in fluoroimmunoassays. Amino-terminal fragment (ATF) of urokinase plasminogen activator (uPA) was expressed in yeast cell (pichia pastoris) to ensure the proper protein folding and purified following the procedure as previously described[1]. All the chemical reagents were used as received without further purification.

Synthesis of Sacrificial $NaGdF_4$:Eu and $NaEuF_4$ Shell Nanoparticles (NPs): In a typical procedure for the synthesis of sacrificial $NaGdF_4$:Eu with an optimized doping concentration of 15% for Eu and $NaEuF_4$ shell NPs, 0.5 mmol of $Ln(CH_3COO)_3 \cdot 4H_2O$ (Ln = Gd and Eu) was added to a 50-mL three-neck round-bottom flask containing 4 mL OA and 8 mL ODE. The mixture was heated at 150 °C under N_2 flow with constant stirring for 30 min to remove the water from the raw materials. After cooling down to 50 °C, 2 mmol of NH_4F and 1 mmol of NaOH dissolved in 5

mL methanol were added and the resultant solution was stirred for 30 min to remove the methanol. Thereafter, the solution was heated to 290 °C under N_2 flow for 30 min and then cooled down to room temperature (RT). The resulting sacrificial NPs were precipitated by addition of acetone, collected by centrifugation at 12000 rpm for 5 min, washed with ethanol for several times, and finally re-dispersed in 10 mL ODE for the preparation of the $NaGdF_4$:Yb/Tm@$NaGdF_4$:Eu@$NaEuF_4$ core-shell-shell (CSS) NPs.

Synthesis of $NaGdF_4$:Yb/Tm@$NaGdF_4$:Eu@$NaEuF_4$ CSS NPs: The synthesis of monodisperse CSS NPs was conducted via a modified successive layer-by-layer injection protocols as previously reported.[2, 3] In a typical procedure, the $NaGdF_4$:Yb/Tm core NPs with optimized doping coencentrations of 20% and 1% for Yb and Tm respectively were first prepared by mixing 0.5 mmol of $Ln(CH_3COO)_3 \cdot 4H_2O$ (Ln = Gd, Yb and Tm,), 4 mL OA and 8 mL ODE in a 100-mL three-neck round-bottom flask followed by heating at 150 °C for 30 min under N_2 flow. After cooling down to 50 °C, 5 mL of methanol solution containing NH_4F (2 mmol) and NaOH (1.25 mmol) was added and stirred for 30 min. Subsequently, the resulting mixture was heated to 300 °C under N_2 flow at a heating rate of 10 °C/min and maintained for 40 min to obtain 13 ± 1.8 nm $NaGdF_4$:Yb/Tm core NPs (Figure S3). After retrieving 1mL of reaction mixture for transmission electron microscopy analysis, 1 mL ODE solution containing calculated amount of 6 ± 1.5 nm $NaGdF_4$:Eu or $NaEuF_4$ sacrificial shell NPs were immediately injected into the reaction mixture and ripened at 300 °C for 15 min followed by the similar injection and ripening cycles. The thickness of the $NaGdF_4$:Eu or $NaEuF_4$ shell layers can be readily tuned by adjusting the injection and ripening cycles. Injection of shell precursors and ripening cycles were performed to a total of 10 cycles to yield monodisperse $NaGdF_4$:20%Yb,1%Tm@$NaGdF_4$:15%Eu@$NaEuF_4$ CSS NPs with optimum upconverting luminescence (UCL) and downshifting luminescence (DSL) of Eu^{3+}. The obtained CSS NPs were precipitated by addition of 30 mL of ethanol, collected by centrifugation at 12000 rpm for 5 min, washed with ethanol several times, and finally re-dispersed in cyclohexane.

Surface Modification of the CSS NPs: To render the hydrophobic CSS NPs hydrophilic and biocompatible, we removed the original OA ligands from their surface by acid treatment as previously reported.[4] In a typical process, 20 mg of the as-synthesized OA-capped CSS NPs were dispersed in 30 mL of acidic ethanol solution (pH 1) and ultrasonicated for 30 min to remove the surface ligands. After the reaction, the NPs were collected by centrifugation at 12000 rpm for 10 min, and further purified by adding an acidic ethanol solution (pH 4). The resulting ligand-free CSS NPs were washed with ethanol and distilled water for several times, and then re-dispersed in distilled water for the following use.

Synthesis of Amine-Functionalized CSS NPs: The surface amine-functionalization of CSS NPs was carried out by using a modified ligand exchange strategy.[5] In a typical process, 5 mL of cyclohexane solution containing 20 mg of OA-capped CSS NPs was mixed with 5 mL of dichloromethane solution of nitrosyl tetrafluoroborate (20 mg) at RT, and gently stirred for 10 min to yield white precipitates, which were collected by centrifugation at 12000 rpm for 4 min, and re-dispersed in 10 mL of DMF to form a transparent solution. Subsequently, 0.2 mmol of AEP was added to the above transparent solution. After stirring for 60 min at RT, the amine-functionalized CSS NPs were precipitated by adding 20 mL of acetone, collected by centrifugation, and washed with DMF and water for several times to remove the excess AEP. The final products were dispersed in distilled water and stored at 4 °C for the following use.

Biotinylation of Ligand-Free CSS NPs: In a typical process, 10 mg of ligand-free CSS NPs were mixed with 20 mg of biotin in 1 mL of distilled water by adding 200 μL of ammonia solution. The mixture solution was allowed for vigorous stirring at 4 °C overnight, and then centrifuged at 14,000 rpm for 10 min to remove the excess biotin. The resulting biotinylated NPs were purified by washing with distilled water for several times, and then dispersed in distilled water and stored at 4 °C.

Quantitative Analysis of Biotin Attached to the Surface of Biotinylated CSS NPs: The amount of biotin attached to the surface of ligand-free CSS NPs can be determined by using the avidin/HABA (4′-hydroxyazobenzene-2-carboxylic acid) reagent.[6] The HABA dye can be attached to avidin to produce a yellow-orange colored complex that absorbs at 500 nm. When the biotinylated sample is mixed with the avidin/HABA complex solution, biotin will displace the HABA dye and cause the absorbance to decrease. In our experiment, the HABA solution was prepared by adding HABA (24 mg) into deionized water (10 mL), followed by the addition of 0.2 mL of NaOH (1 M). The undissolved HABA particulates were removed by filtration. The avidin/HABA complex solution was prepared by dissolving avidin (5 mg) in 50 mL of phosphate buffered saline (PBS), followed by the addition of 0.3 mL of HABA solution. 1 mg of biotinylated CSS NPs for avidin/HABA assay was dissolved in 0.03 mL of PBS (50 mM, pH=7.1) and mixed with 0.3 mL of avidin/HABA solution. The amount of biotin in the biotinylated CSS NPs was determined according to the calibration curve generated by adding a known amount of biotin in the avidin/HABA solution sequentially, followed by recording the absorbance at 500 nm (OD_{500}).

Preparation of the Enhancer Solution: The enhancer solution was prepared by using a modified protocol as previously reported.[7] In a typical procedure, β-NTA and TOPO with final concentrations of 15 μM and 50 μM, respectively, were mixed in 100 mL of aqueous solution (pH 2.3) containing 1 % (m/v) acetate and 0.1 % (m/v) Triton X-100.

UCL and DSL Bioassays of AFP by Using the Biotinylated CSS NPs: After anti-AFP monoclonal antibody (diluted to 10 μg/mL with 0.1 M carbonate buffer of pH 9.6) was coated on the wells (100 μL per well) of a 96-well Nunc Immobilizer Amino plate via incubation at 37 °C for 1 h, 300 μL of blocking solution (100 mM carbonate buffer containing 0.1% of 2-aminoethanol, pH 9.6) was added in each well and incubated at 37 °C for another 1 h to block other free-standing groups on the plate. The plate was washed with PBST (Phosphate Buffered Saline containing 150 mM of NaCl and 0.05% (v/v) TWEEN 20, pH 7.2) for 3 times. Thereafter, different amounts of standard solution of human AFP antigen dissolved in 100 μL of phosphate buffer solution was added to each well, and then incubated at 37 °C for 1 h for coupling with AFP antigen, after which the plate was aspirated and washed with PBST for 3 times. Subsequently, the biotinylated anti-AFP monoclonal antibody (2 μg/mL, 100 μL per well) was added to each well and the plate was incubated at 37 °C for 1 h followed by washing with PBST for 3 times. Afterwards, avidin (10 μg/mL, 100 μL per well) was added to each well, allowed for incubation at 37 °C for 45 min and washed with PBST for 3 times. Thereafter, the biotinylated CSS NPs (20 μg/mL, 100 μL per well) was added to each well and the plate was incubated at 37 °C for 60 min, during which the biotinylated CSS NPs conjugated with biotinylated AFP antibody through a specific binding between avidin and biotin. After washing with PBST for 6 times, the plate was subjected to in-site UCL detection on a self-made UCL biodetection system coupled with a multimodal microplate reader (Synergy 4, BioTeK) at RT. Thereafter, 300 μL of the enhancer solution was added to each well followed by slight shaking for 4 min to yield the highly luminescent β-NTA-Eu^{3+}-TOPO complex, after which the plate was subjected to time-resolved PL detection on the multimodal microplate reader at room temperature, by setting the delay time and gate time to be

250 μs and 2 ms, respectively. Wherein, the excitation and emission wavelengths were set to be 340 nm and 615 nm, respectively. For comparison, control experiments by replacing AFP with BSA were also conducted under otherwise identical conditions. Every measurement was repeated for four times to yield the average value and deviation. The assay of human serum samples was conducted following the same procedure by simply replacing the human AFP standard solution with human serum samples. The AFP levels in human serum samples were determined according to the calibration curve derived from the assay of human AFP standard solution.

AFP Assay by Using the Commercial DELFIA Kit: The AFP levels in human serum samples were determined following a modified protocol of commercial DELFIA kit for AFP (Daan Gene Co., Ltd. of Sun Yat-Sen University, China). In brief, after anti-AFP monoclonal antibody (diluted to 10 μg/mL with 0.1 M carbonate buffer of pH 9.6) was coated on the wells (100 μL per well) of a 96-well Nunc Immobilizer Amino plate via incubation at 37 °C for 1 h, 300 μL of blocking solution was added in each well and incubated at 37 °C for another 1 h to block other free-standing groups on the plate. The plate was washed with PBST (Phosphate Buffered Saline containing 150 mM of NaCl and 0.05% (v/v) TWEEN 20, pH 7.2) for 3 times. Human AFP standard solution (100 μL per well) was then added to each well. After incubation at 37 °C for 1 h, the wells were washed with PBST for 3 times. Then the Eu^{3+}-DTTA-labeled anti-AFP antibody (10 μg/mL, 100 μL per well) was added to each well and the plate was incubated at 37 °C for 1 h. After washing with PBST for 6 times, the enhancer solution (300 μL) was added into the plate, and then the plate was subjected to for time-resolved photoluminescence (TRPL) detection on the multimodal microplate reader at RT. The excitation and emission wavelengths were set to be 340 nm and 615 nm, respectively. The delay time and gate time were 250 μs and 2 ms, respectively. Every measurement was repeated for four times to yield the average value and deviation. The assay of human serum samples was conducted following the same procedure by simply replacing the human AFP standard solution with human serum samples. The AFP levels in human serum samples were determined according to the calibration curve derived from the assay of human AFP standard solution.

Bioconjugation of Amine-Functionalized CSS NPs with ATF: The bioconjugation of amine-functionalized CSS NPs with ATF of urokinase plasminogen activator (uPA) was carried out by following the procedure we previously reported.[8] Briefly, 5 mg of ATF and 10 mg of o-Benzotriazole-N,N,N',N'-tetramethyl-uronium-hexafluoro-phosphate (HBTU) were firstly dissolved in a solution of 0.9 mL DMF and 0.1 mL N,N-diisopropylethylamine (DIEA). After the activation of ATF at RT for 30 min, 20 mg of amine-functionalized CSS NPs was added to the above solution and sonicated for 5 min. The mixture solution was then allowed to shake gently overnight at 4 °C. Finally, the ATF-coupled CSS NPs were obtained by centrifugation, washed with DMF and distilled water for several times. The resulting ATF-coupled CSS NPs were dispersed in water and stored at 4 °C for targeted cancer cell imaging.

Cell Culture and confocal laser scanning microscopy (CLSM) Imaging *in-Vitro*: Human lung cancer cells (H1299) and human embryo lung fibroblast (HELF) cell lines were routinely maintained in RPMI-1640 (GIBCO BRL), supplemented with 10% (v/v) heat-inactivated fetal calf serum, penicillin (100 U mL^{-1}), and streptomycin (100 U mL^{-1}) at 37 °C under humidified air containing 5% CO_2. Cells were seeded into culture plates and allowed to adhere for 24 h. After washing with phosphate buffered saline (PBS), the cells were incubated in PBS buffer containing 0.5 mg/mL ATF-coupled CSS NPs at 37 °C for 2 h under 5% CO_2, and then washed with PBS sufficiently to remove excess CSS NPs. The cells were subsequently incubated with 4',6-diamidino-2-phenylindole (DAPI) at RT for 5 min and washed with PBS for several times. The cell imaging was performed by using CLSM equipped with an Olympus FV1000

scanning unit. Cells were excited by a 980-nm laser, and the UCL signals were detected in the red channel (600-650 nm) and blue channel (450-490 nm), respectively.

Dark Cytotoxicity and Phototoxicity of ATF-Coupled CSS NPs: The dark cytotoxicity of ATF-coupled CSS NPs was tested by using a Cell Counting Kit-8 (CCK-8) assay on the human embryo lung fibroblast (HELF) cells. In brief, HELF cells were seeded into a 96-well cell culture plate at a density of 2×10^4/well and cultured at 37 °C under humidified air containing 5% CO_2 for 24 h before the addition of different concentrations of ATF-coupled CSS NPs (0, 31.25, 62.5, 125, 250, 500 and 1000 μg/mL, diluted in RPMI 1640) into the wells. CCK-8 was subsequently applied to the cells followed by an incubation at 37 °C under 5% CO_2 for 4 h. The optical density at 450 nm (OD_{450}) of each well was measured on a multimodal microplate reader (Synergy 4, BioTek). The inhibition rate of cell growth was calculated by the following formula: Cell viability (%) = (mean of absorbance value of treatment group/mean of absorbance value of control) × 100%. The same protocol was utilized to determine the phototoxicity of ATF-coupled CSS NPs, except that the HELF cells were irradiated by using a 980-nm laser for 2 min after the incubation of ATF-coupled CSS NPs with HELF cells for 4 h.

Real-Time Cytotoxicity Assay of ATF-Coupled CSS NPs Based on Electric Cell-Substrate Impedance Sensing: The real-time cytotoxicity of ATF-coupled CSS NPs was monitored by means of electric cell-substrate impedance sensing (ECIS) measurement. An 8-well plate (8W10E, Applied Biophysics, NY) was used in ECIS. Each well has a surface area of 0.8 cm^2 and holds 600 μL of culture medium, which contains ten individual interdigitated gold microelectrodes (diameter of 250 μm) and connected to an ECIS Z system (Applied Biophysics, Inc., NY, USA) to measure electric current flow through the medium. The frequency of electric impedance response for each cell type was optimized with multi-frequency optimization run with a sampling interval of 60 s. In a typical ECIS experiment, the 8W10E plates were equilibrated with cell medium in an incubator (37 °C, 100% humidity and 5% CO_2) for at least 2 h before the cells (2×10^5 per well) were seeded into each well of the array plates with fresh medium (200 μL). The plates were then connected to ECIS instrument and put back into the incubator. The cells were allowed to attach to the plates for 5 h until the electric impedance response of cells on the plates was stabilized and became a straight line. Thereafter, the ECIS measurement was paused and the culture medium of wells in the 8W10E plates was replaced with the medium containing ATF-coupled CSS NPs at a final concentration of 1 mg/mL (or DOX at a final concentration of 100 μg/mL as a negative control or with fresh medium as a positive control). Thereafter, the measurement was restarted and the electric impedance data collection was continued throughout the incubation and extended for 20 h after the administration.

Characterization: Transmission electron microscopy (TEM), high angle annular dark-field scanning TEM (HAADF-STEM), electron energy loss spectroscopy (EELS) and the elemental 2D-mapping measurements were conducted by using TECNAI G2F20 TEM. The ζ-potentials and hydrodynamic diameter distribution for the ligand-free and biotinylated CSS NPs dispersed in distilled water (pH 6.9) were determined by means of dynamic light scattering measurement (Nano ZS ZEN3600, Malvern). DSL emission/excitation spectra and decays were recorded on a spectrometer equipped with both continuous (450 W) xenon and pulsed flash lamps (FLS980, Edinburgh Instruments). UCL spectrum was measured upon 980-nm NIR excitation from a continuous-wave diode laser. UCL excitation spectrum and UCL decays were measured with a customized UV to mid-infrared steady-state and phosphorescence lifetime spectrometer (FSP920-C, Edinburgh Instrument) equipped with a digital oscilloscope (TDS3052B, Tektronix) and a tunable mid-band Optical Parametric Oscillator (OPO) pulse laser as the excitation source (410-2400 nm, 10 Hz,

pulse width 5 ns, Vibrant 355II, OPOTEK). The absolute UCL quantum yield (QY) for the CSS NPs was measured with a customized UCL spectroscopy system at room temperature upon a 980-nm diode laser excitation at a power density of 100 W/cm^2, and the UCL peaks from Eu^{3+} and Tm^{3+} ions in the spectral range of 270-850 nm were integrated for the QY determination. All the spectral data collected were corrected for the spectral response of both the spectrometer and the integrating sphere. UCL and DSL bioassays of AFP was conducted on our customized UCL biodetection system integrated with a microplate reader (Synergy 4, BioTek). Confocal imaging of cells was performed with a modified Olympus FV1000 laser scanning confocal microscope (60 × oil-immersion objective lens).

II. Supplementary Tables

Table S1. Comparison of PL signals of biotinylated CSS NPs (20 μg/mL) dispersed in water and their counterparts dissolved in the enhancer solution upon UV excitation at 273 and 340 nm, respectively.

	CSS NPs dispersed in H_2O	The dissolved counterparts in the enhancer solution diluted by a factor of					
		10	100	10^3	10^4	10^5	10^6
PL signals (Counts)	2440	overflow	overflow	overflow	67547	10090	2143

Table S2. Comparison of the AFP levels in 20 human serum samples (from normal humans with AFP level lower than 20 ng/mL and cancer patients with AFP level higher than 20 ng/mL) independently determined by the UCL and DSL bioassays based on the CSS NPs and the commercial DELFIA kit using Eu^{3+}-DTTA complex, respectively. Data represent the (mean ± standard deviation) of three independent experiments. The unit of the AFP levels is ng/mL.

**Samples**	**UCL**	**DSL**	**DELFIA**	**Samples**	**UCL**	**DSL**	**DELFIA**
1	69.74 ± 7.25	67.45 ± 6.98	76.14 ± 2.39	11	5.19 ± 1.46	5.72 ± 2.11	6.13 ± 0.59
2	49.48 ± 4.46	56.84 ± 7.51	55.22 ± 2.20	12	19.69 ± 2.49	16.22 ± 3.22	19.62 ± 0.89
3	24.83 ± 1.92	2.66 ± 1.67	28.31 ± 0.53	13	16.60 ± 0.43	18.11 ± 0.54	12.55 ± 0.29
4	28.89 ± 3.06	25.37 ± 3.18	32.10 ± 1.23	14	14.43 ± 0.82	10.63 ± 2.45	15.44 ± 1.37
5	29.10 ± 2.38	20.90 ± 3.49	29.94 ± 1.76	15	10.90 ± 0.91	12.37 ± 0.36	11.23 ± 0.47
6	10.57 ± 1.29	8.80 ± 0.58	9.89 ± 0.45	16	141.2 ± 9.66	137.7 ± 8.71	155.5 ± 2.44
7	17.70 ± 0.63	16.49 ± 1.82	19.70 ± 0.16	17	19.90 ± 1.27	20.82 ± 2.46	21.86 ± 0.63
8	0.98 ± 0.32	0.85 ± 0.43	1.03 ± 0.08	18	31.33 ± 0.77	26.32 ± 2.11	32.20 ± 0.82
9	15.79 ± 1.31	13.24 ± 2.44	12.90 ± 0.47	19	8.55 ± 3.22	8.85 ± 2.78	11.42 ± 1.27
10	13.07 ± 1.53	8.95 ± 3.54	13.66 ± 1.03	20	9.35 ± 0.41	11.44 ± 2.90	9.56 ± 0.38

III. Supplementary Figures

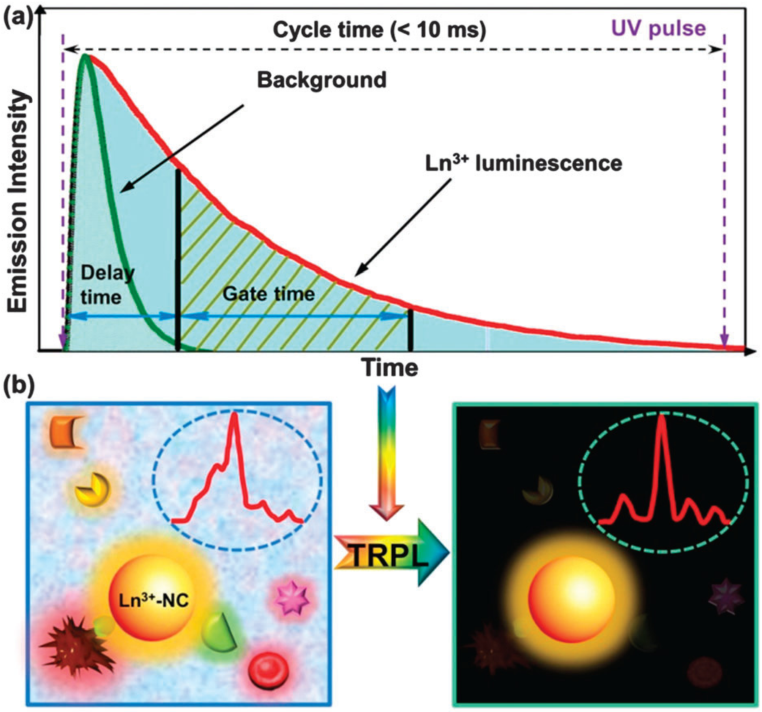

Figure S1. (a) The principle for TRPL bioassay and (b) schematic illustration of trivalent lanthanide (Ln^{3+})-doped nanoprobes for background-free TRPL biodetection.[9] Conventional luminescent bioassay, by means of steady-state detection, often suffers from the interferences from short-lived scattered light and other autofluorescence of biological samples or assay plates, which results in a low signal-to-noise ratio (S/N) and thus limited detection sensitivity (Figure S1b, left). To completely eliminate such negative interferences and improve the detection sensitivity, the technique of TRPL, by utilizing the long-lived luminescence of Ln^{3+} in the compounds such as commercial lanthanide-chelating agents, has been introduced in biomedical analyses. In this way, the short-lived background luminescence can be suppressed when measuring the long-lived PLof Ln^{3+} by setting appropriate delay time and gate time, which thereby provides a signal with remarkably high S/N (Figure S1b, right). More importantly, because data sampling in the TRPL detection is completed in a few milliseconds, it can be repeated several thousand times during a minute, leading to a very high sensitivity within a few minutes.

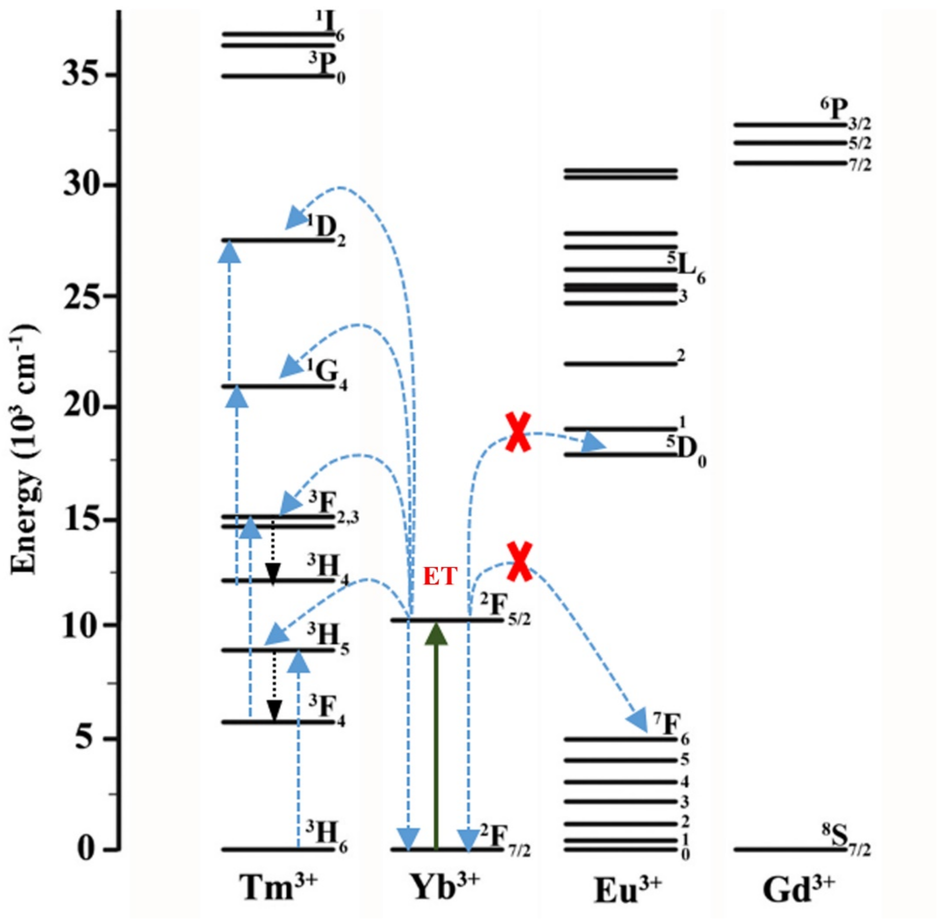

Figure S2. Partial energy level diagram in the range from 0 to 37000 cm^{-1} for the Ln^{3+} ions (Ln = Eu, Gd, Tm, Yb). Due to large energy mismatch (~5000 or 7000 cm^{-1}) between the $^2F_{5/2}\rightarrow{}^2F_{7/2}$ transition of Yb^{3+} and the $^7F_0\rightarrow{}^7F_6$ (or $^5D_0$) transition of Eu^{3+}, the direct Yb^{3+}-to-Eu^{3+} energy transfer involved in the upconversion processes becomes inefficient for the Yb^{3+} and Eu^{3+} co-doped NPs. It differs significantly from the cases of Yb^{3+} and Tm^{3+} (or Er^{3+}) co-doped upconversion NPs that are highly efficient. As a result, it is notoriously difficult to achieve efficient upconversion emission of Eu^{3+} via conventional approaches by simply co-doping Yb^{3+} in NPs.

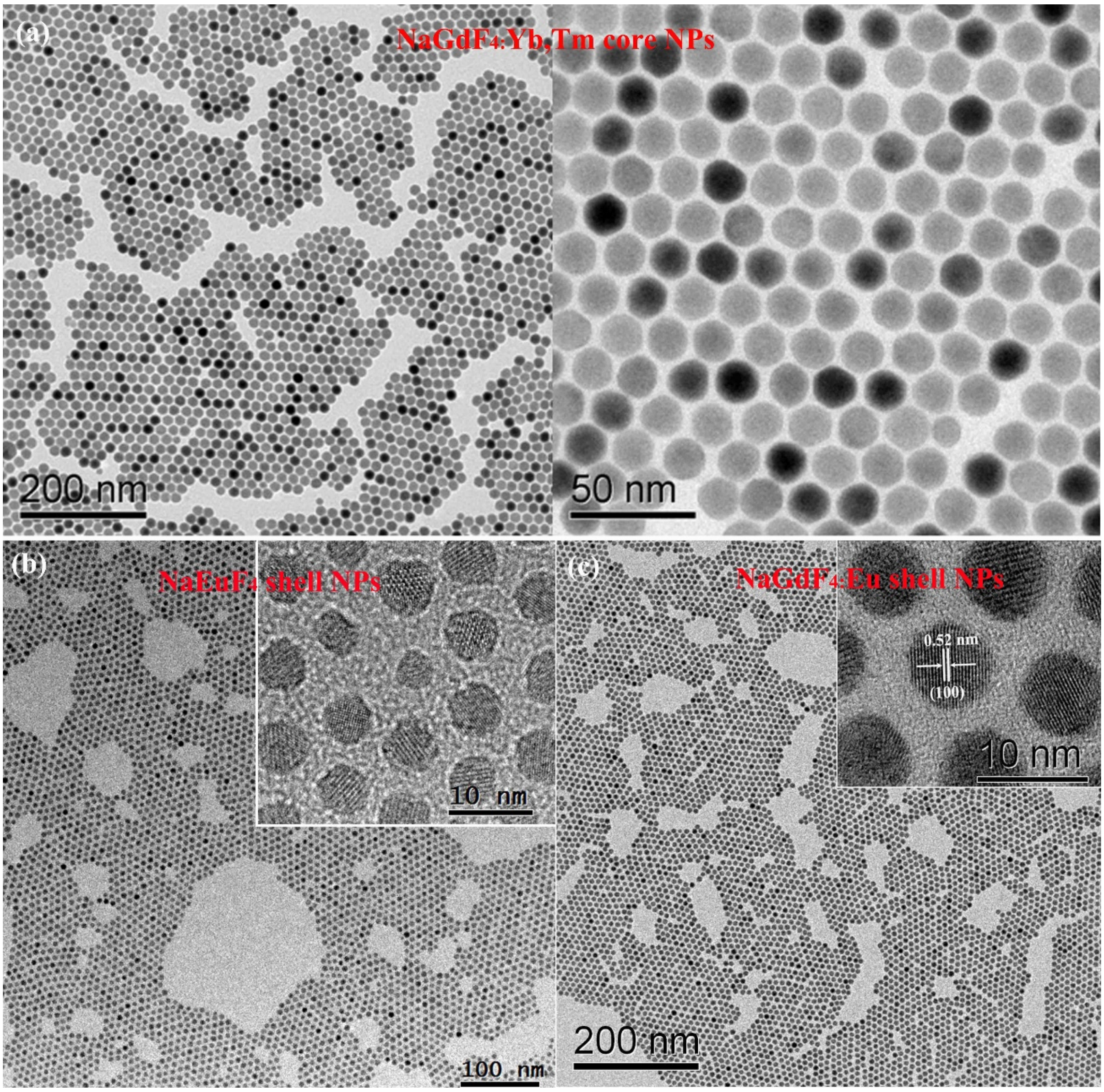

Figure S3. TEM images for the (a) $NaGdF_4$:20%Yb,1%Tm core, sacrificial (b) $NaEuF_4$ and (c) $NaGdF_4$:15%Eu shell NPs at different magnifications. The average particle sizes were estimated to be 13 ± 1.8, 6 ± 1.5 and 6 ± 1.5 nm from TEM images for the $NaGdF_4$:20%Yb,1%Tm core, sacrificial $NaGdF_4$:15%Eu and $NaEuF_4$ shell NPs, respectively.

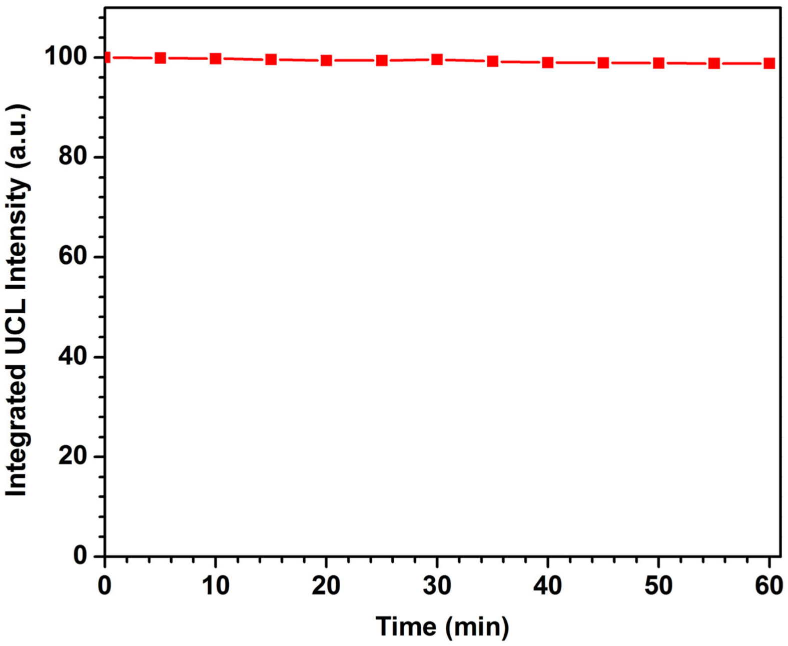

Figure S4. UCL intensity for the CSS NPs dispersed in cyclohexane as afunction of the 980-nm irradiation time. It was observed that the UCL intensity for the CSS NPs remained essentially unaltered after continuous 980-nm irradiation for 60 min (radiation density of ~10 W/cm^2), indicative of the superior photostability of the CSS NPs we obtained.

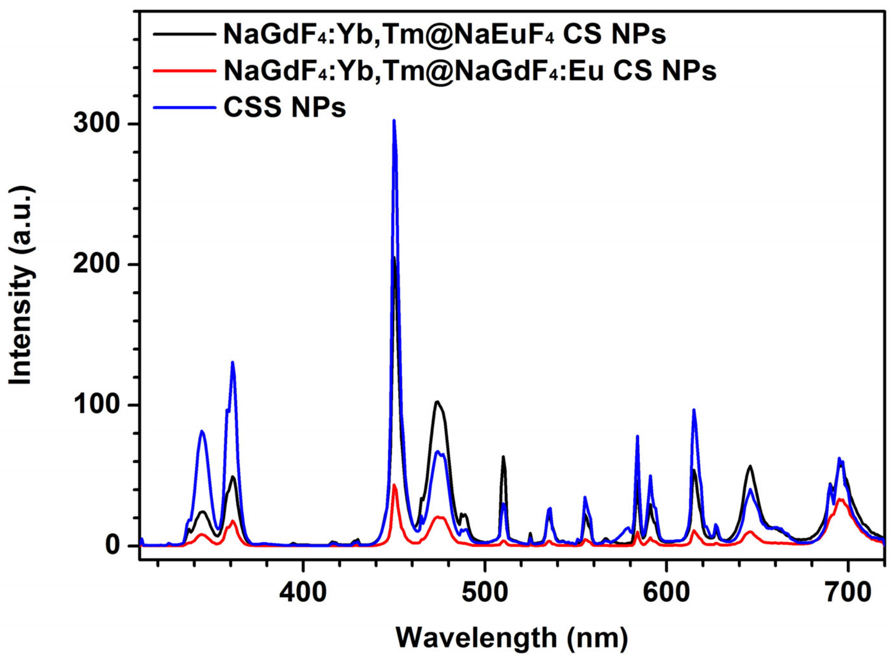

Figure S5. Comparison of UCL intensities for the CSS NPs and their $NaGdF_4$:20%Yb/1%Tm@$NaGdF_4$:15%Eu and $NaGdF_4$:Yb/Tm@$NaEuF_4$ core-shell (CS) counterparts. It was found that the UCL intensity for the CSS was about 2.6 and 1.7 times stronger than those of the $NaGdF_4$:Yb/Tm@$NaGdF_4$:Eu and $NaGdF_4$:Yb/Tm@$NaEuF_4$ CS counterparts.

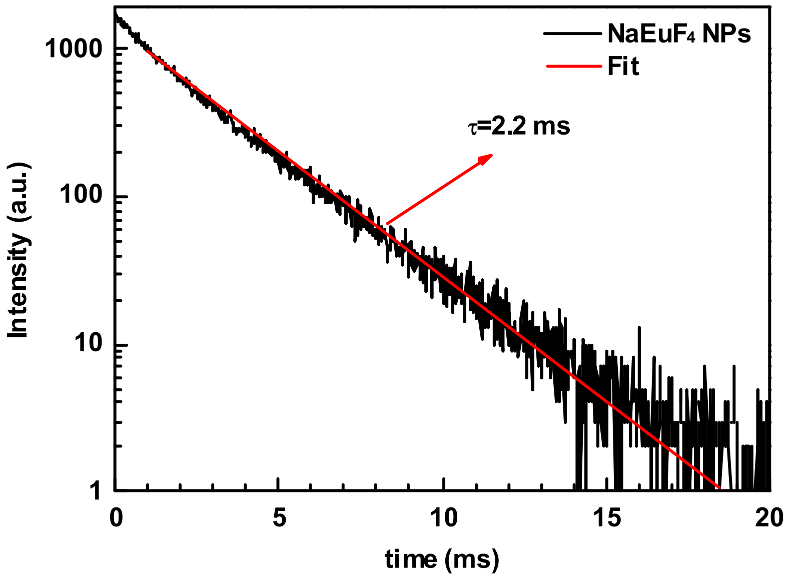

Figure S6. DSL decay from $^5D_0$ of Eu^{3+} in $NaEuF_4$ NPs (~6 nm) by monitoring the Eu^{3+} emission at 615 nm. By fitting with a single-exponential function, the PL lifetime of Eu^{3+} in $NaEuF_4$ NPs was determined to be 2.2 ms.

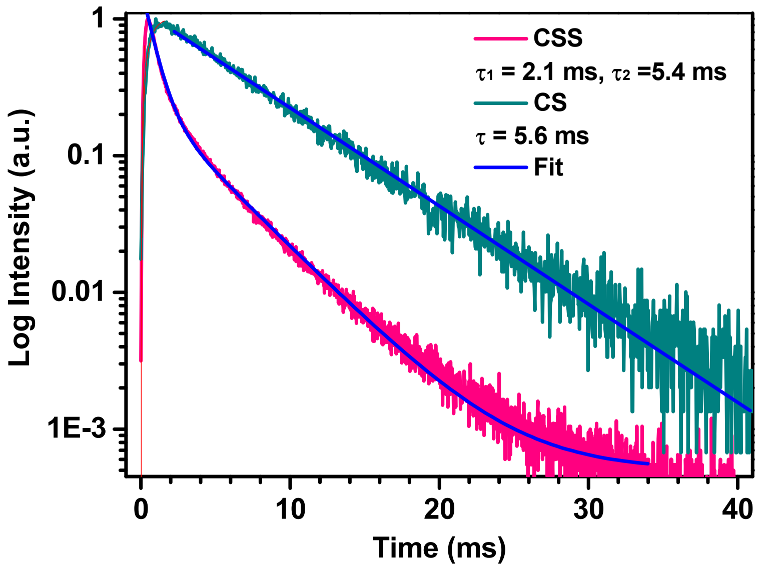

Figure S7. UCL decays from $^5D_0$ of Eu^{3+} in the CS and CSS NPs by monitoring the Eu^{3+} UC emission at 615 nm upon excitation by a 980-nm pulsed laser. Note that the UCL decay curve of Eu^{3+} in the CS NPs was fitted with single exponential function, while the UC decay curve of Eu^{3+} in the CSS NPs was fitted with double exponential function.

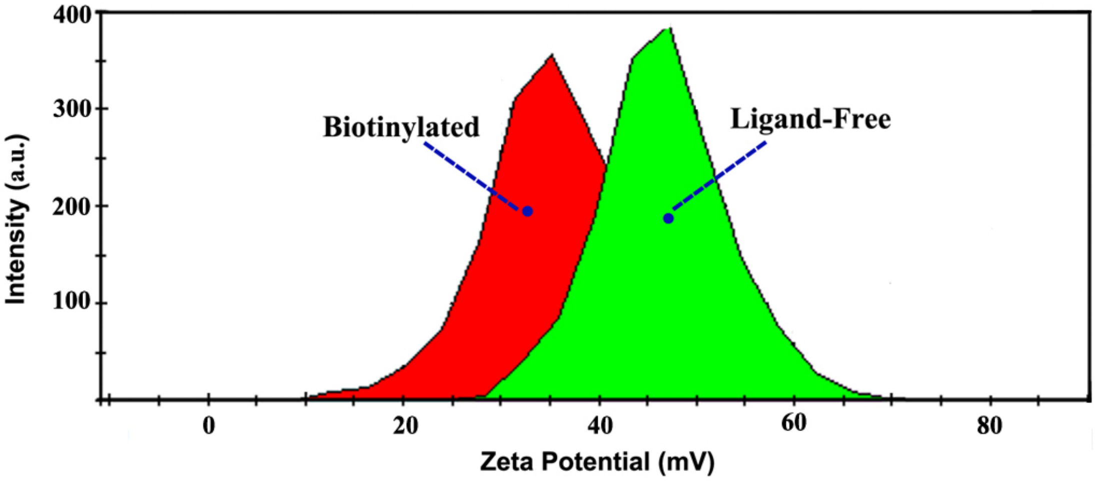

Figure S8. The ζ-potentials for the ligand-free and biotinylated CSS NPs obtained from the dynamic light scattering measurement. The ζ-potential for the ligand-free CSS NPs when dispersed in aqueous solution (pH 6.9) was determined to be +46.7 mV, indicating the positively charged Eu^{3+} ions exposed on the surface of ligand-free CSS NPs. The ζ-potential for the CSS NPs was observed to change from +46.7 mV to +32.9 mV, as a result of reduced positively charged Eu^{3+} ions exposed on their surface, thus confirming the successful conjugation of biotin onto the surface of CSS NPs.

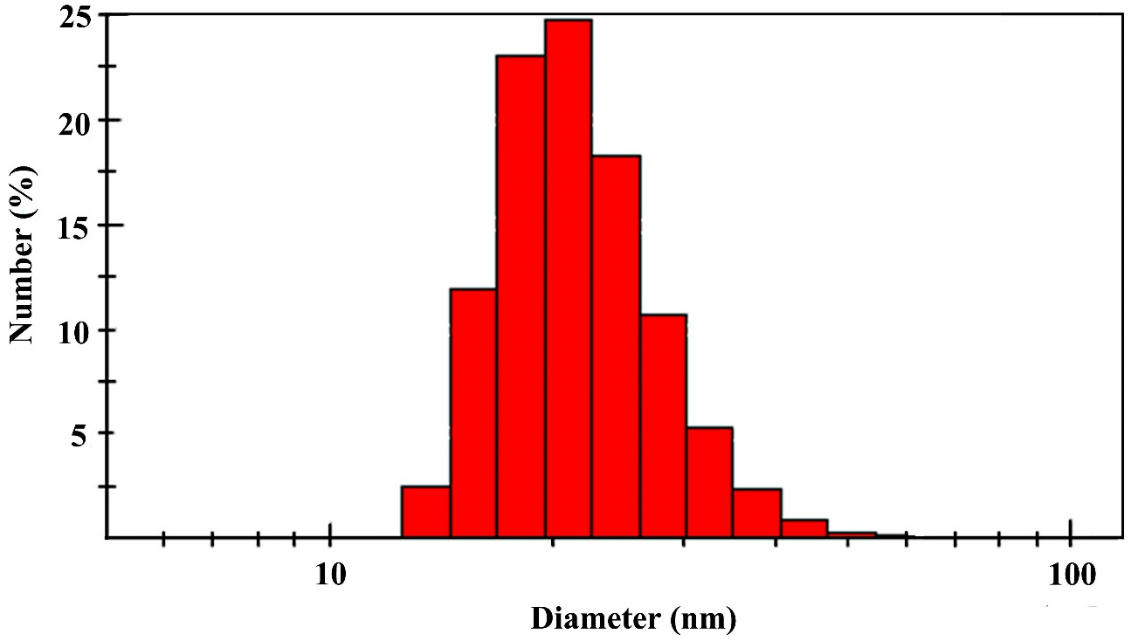

Figure S9. Hydrodynamic diameter distribution of the ligand-free CSS NPs. The hydrodynamic diameter for the ligand-free CSS NPs was determined to be 22 ± 1.9 nm, which is basically in agreement with their original OA-capped counterparts, indicating that the removal of surface ligands has no noticeable influence on the size of the CSS NPs.

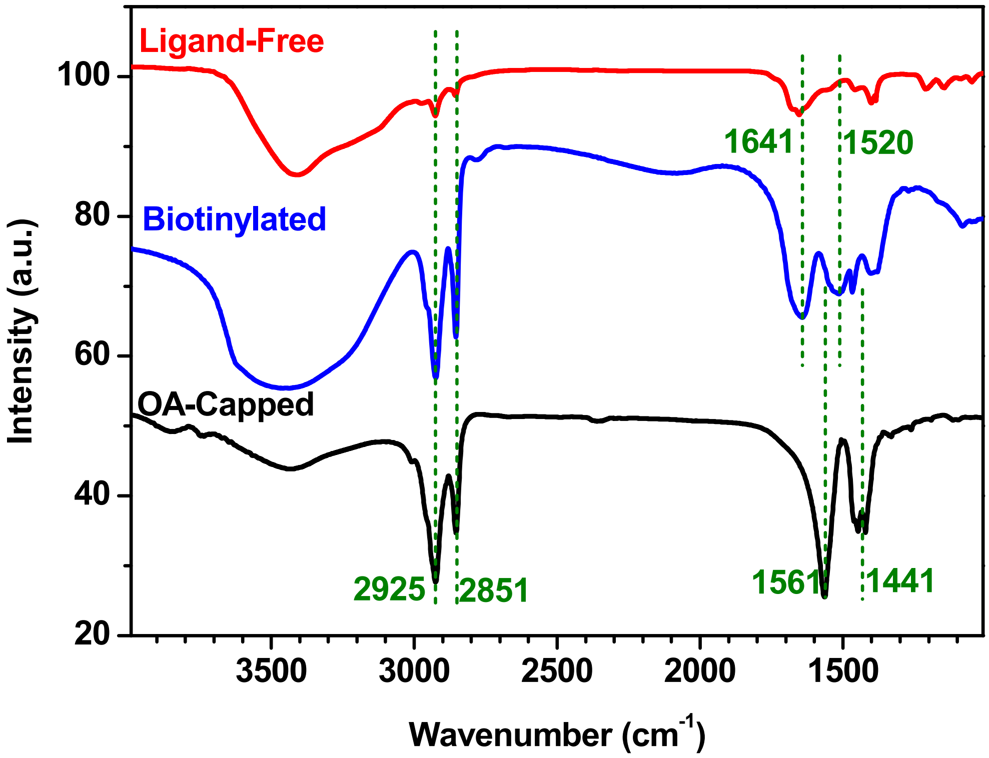

Figure S10. FTIR spectra for the OA-capped, ligand-free and biotinylated CSS NPs. In comparison with the OA-capped NPs, the original asymmetric and symmetric stretching vibrations of methylene ($-CH_2-$) in the long alkyl chain peaking at 2925 and 2851 cm^{-1} and the asymmetric and symmetric stretches of carboxyl (-COO-) peaking at 1561 and 1441 cm^{-1} disappeared for the ligand-free NPs, indicating the successful removal of OA ligands from the surface of CSS NPs. The biotinylated CSS NPs exhibited strong amide bands centered at 1641 and 1520 cm^{-1} and enhanced stretching vibrations of methylene ($-CH_2-$) relative to those of ligand-free NPs, thus verifying the successful conjugation of biotin onto the surface of CSS NPs.

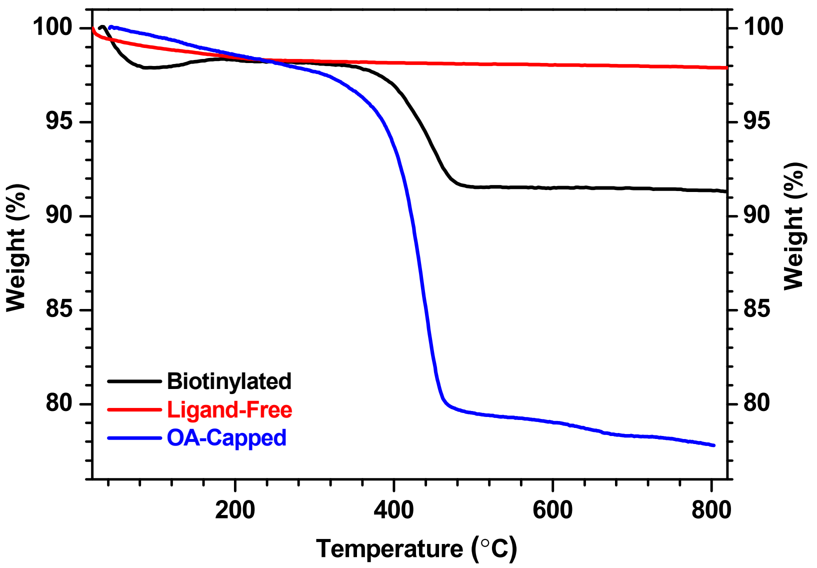

Figure S11. Thermogravimetric analysis curves for the OA-capped, ligand-free, and biotinylated CSS NPs under N_2 atmosphere in the temperature range of 30-800 °C at a rate of 10 °C/min. The OA-capped NPs display a large weight loss of ~22% with a decomposition temperature of 200 °C. For comparison, the ligand-free counterparts show a much smaller weight loss of ~2% and no obvious decomposition temperature, which unambiguously verifies the successful removal of the OA ligand from the surface of the NPs. By contrast, the biotinylated NPs exhibit a weight loss of ~8% with a clear decomposition temperature of ~380 °C, due to the conjugation of biotin onto the surface of the ligand-free NPs.

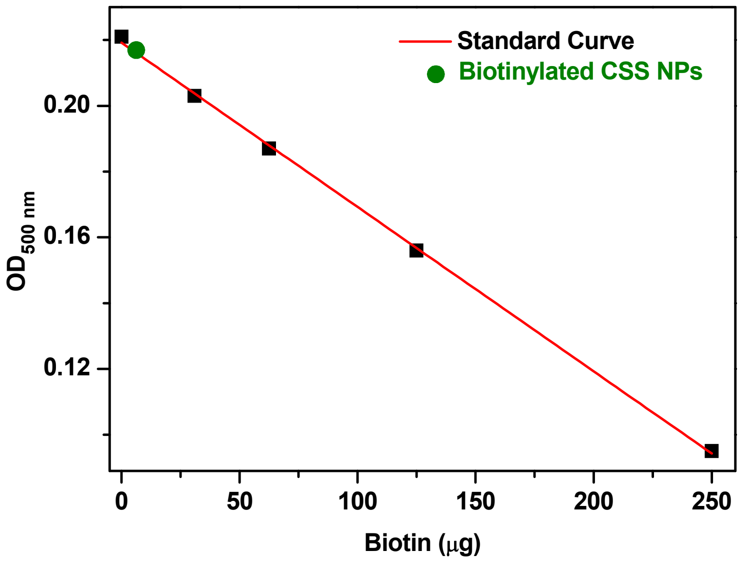

Figure S12. Quantitative analysis of the biotin content in biotinylated CSS NPs. By using an avidin/HABA reagent, the amount of biotin attached to the NPs was determined to be 1.2 μg/mg, and the number of biotin molecule per NP was calculated to be 8.1.

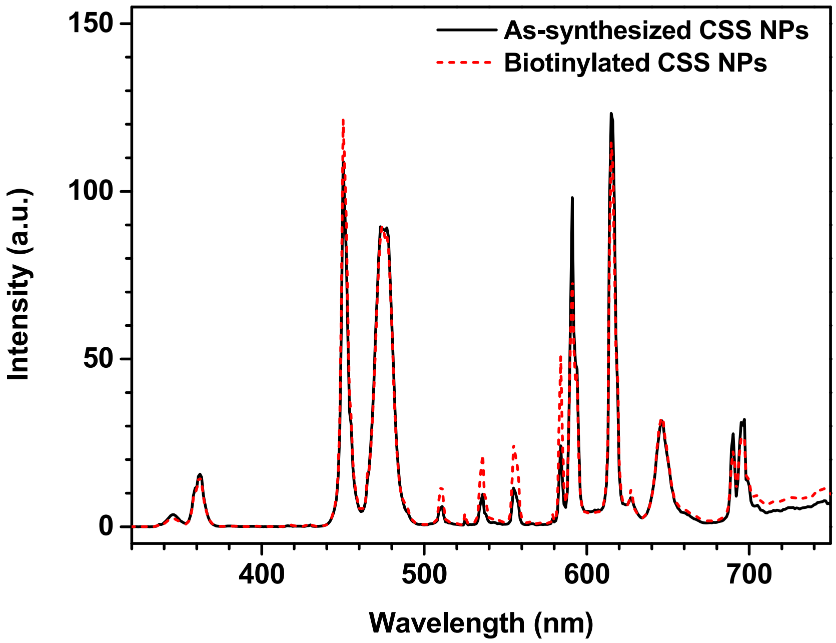

Figure S13. Comparison of UCL for the as-synthesized CSS NPs dispersed in cyclohexane and the biotinylated CSS NPs dispersed in water with a concentration of 0.5 mg/mL upon NIR excitation at 980 nm. It can be seen that the UCL for the biotinylated CSS NPs in water remain nearly unchanged with their parent NPs in cyclohexane due to the effective protection of the outermost $NaEuF_4$ shell against surface quenching from the attached biotin and solvent water, indicating the excellent UCL performance of the Eu^{3+}-activated red luminescent bioprobes we developed.

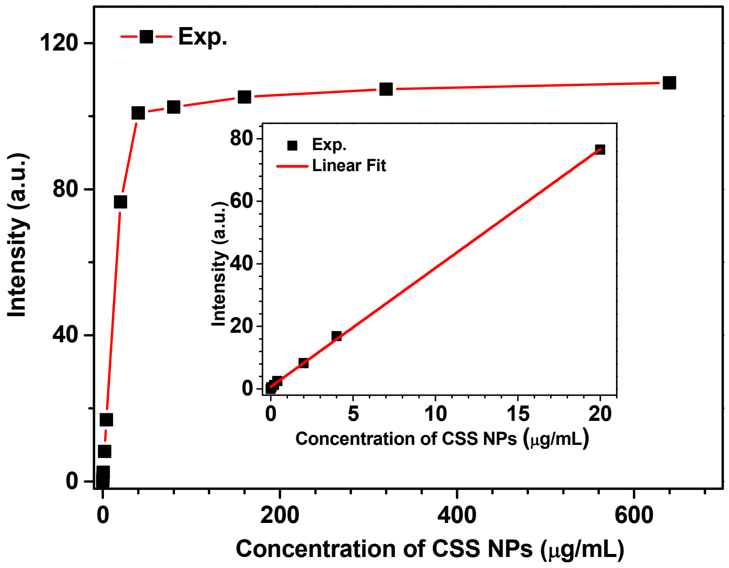

Figure S14. Concentration-dependent dissolution-enhanced PL signals of biotinylated CSS NPs dissolved in the enhancer solution (300 μL). The inset shows the linear range of the dissolution-enhanced PL signal versus the CSS NP concentration (0-20 μg/mL). It was observed that the red DSL signal of Eu^{3+} increased linearly with the CSS NPs concentration in the large range of 0-20 μg/mL, which is highly desirable to ensure reproducible and accurate detection results of tumor markers in clinical bioassays.

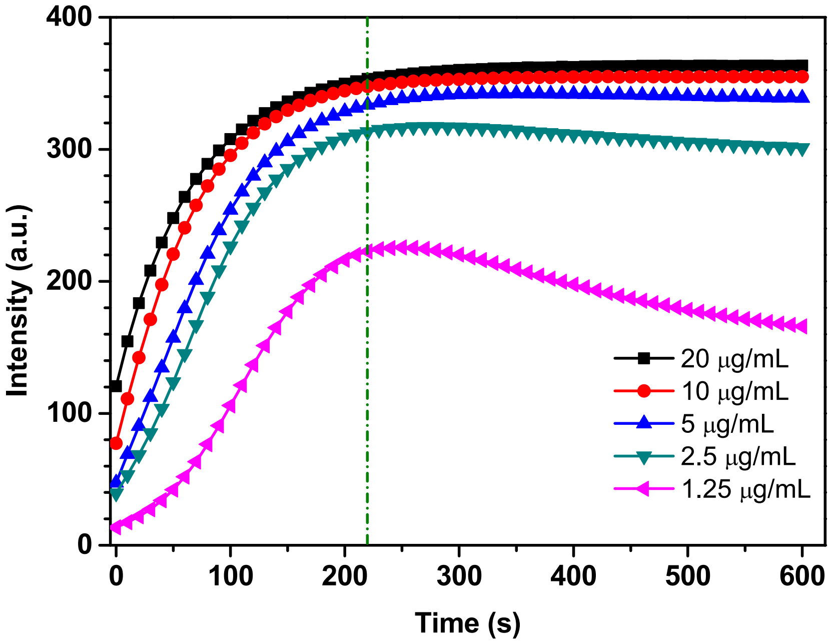

Figure S15. Time-dependent PL signals of the biotinylated CSS NPs with different concentrations upon addition of the enhancer solution (300 μL). It was found that even for biotinylated CSS NPs with a concentration as high as 20 μg/mL, its dissolution-enhanced PL signal reached a plateau within 4 min upon addition of the enhancer solution, thus revealing the fast PL response of our nanoprobes. Meanwhile, the PL intensity for the biotinylated CSS NPs with a concentration less than 2.5 μg/mL was observed to decrease after 4 min upon addition of the enhancer solution, which is caused by the simultaneous dissociation of Gd^{3+} from the CSS NPs.

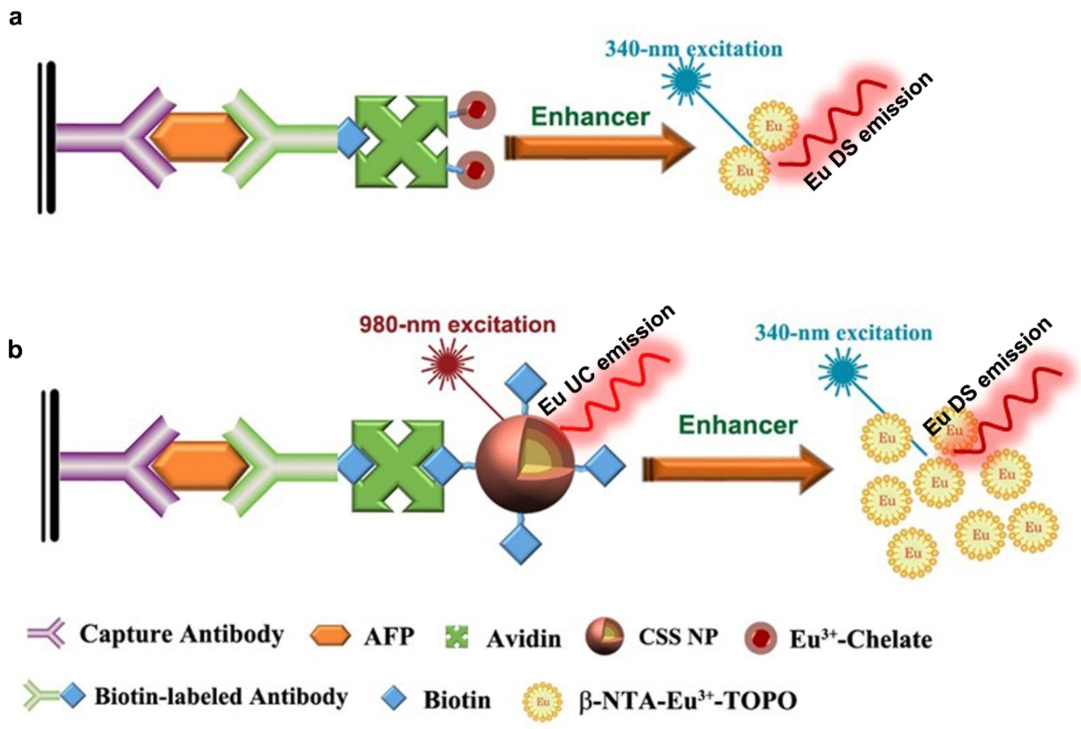

Figure S16. Schematic illustration of (a) conventional commercial DELFIA and the proposed (b) UCL and DSL bioassays based on the biotinylated CSS NPs, where the anti-AFP antibody, AFP antigen, and biotinylated anti-AFP antibody were selected as the capture antibody, analyte and detection antibody, respectively. Conventional DELFIA uses Ln^{3+}-chelates as molecular bioprobes in a classical heterogeneous sandwich-type bioassay, which generates only a few β-NTA-Eu^{3+}-TOPO complex for TRPL measurement due to the low Ln^{3+} labeling ratio of the Ln^{3+}-chelates (up to 10-30 Ln^{3+} labels per biomolecule). By contrast, the DSL bioassay employs the CSS NPs as nanoprobes. Benefiting from concentrated Eu^{3+} ions in single CSS NP (~7000 per CSS NP), much more highly luminescent β-NTA-Eu^{3+}-TOPO complex are formed upon addition of the enhancer solution, which significantly amplifies the TRPL signal and thus improves the detection sensitivity as compared to that of conventional DELFIA. The UCL bioassay makes use of the NIR excitation at 980 nm, where the biological cells and tissues have minimal absorption thus no auto-fluorescence is produced in the detection, thereby providing a much better dimension to enhance the detection sensitivity of AFP.

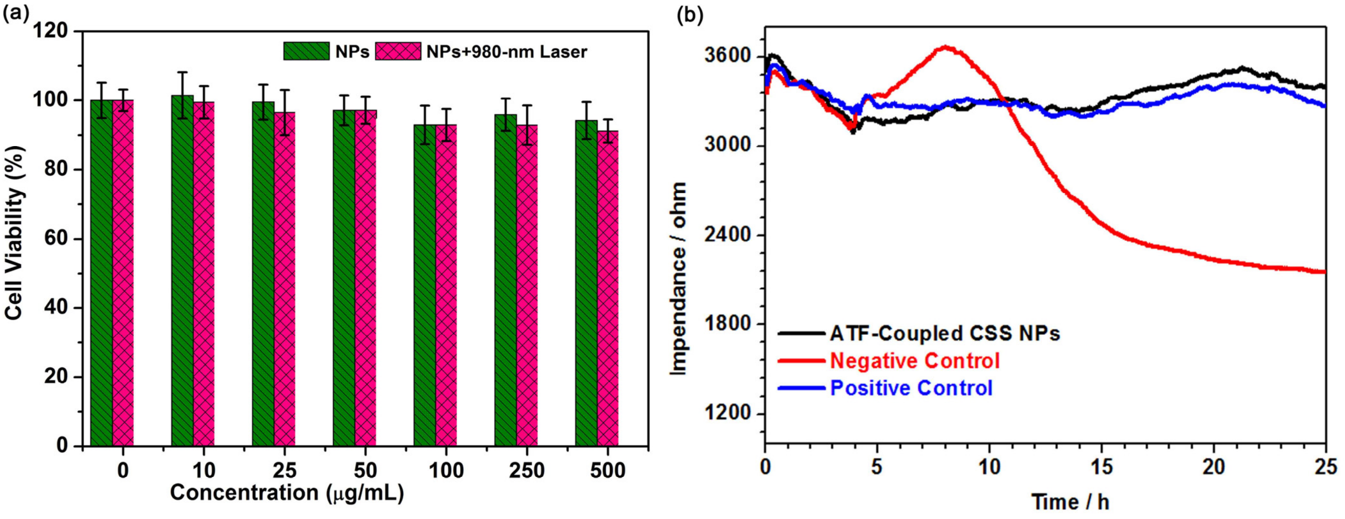

Figure S17 (a) In-vitro dark cytotoxicity (green) and phototoxicity (pink and orange) of ATF-coupled CSS NPs against HELF cells after 2 h incubation by using CCK-8 assay. The cell viability was determined to be larger than 90% even at a concentration as high as 500 μg/mL for ATF-coupled CSS NPs either in the dark or 980-nm light irradiation for 2 min. (b) In-vitro real-time cytotoxicity of ATF-coupled CSS NPs against HELF cells after administration by means of electric cell-substrate impedance sensing (ECIS) technique. Black line represents the impedance of HELF cells incubated with 0.5 mg/mL of ATF-coupled CSS NPs, while the blue and red lines are the impedance of HELF cells for the positive (incubated with fresh medium) and negative (incubated with 100 μg/mL doxorubicin (DOX) control experiments, respectively. ECIS, more sensitive than CCK-8 assay, is a label-free approach to follow the cytotoxic response of adherent cells in real-time based on electric impedance of cells grown on gold-film electrodes, where dead cells detached from gold electrode should not contribute to impedance, and therefore, the measured electric impedance is proportional to the number of live cells attached to the gold electrode. For comparison, both positive control experiment by using fresh HELF cells and negative control experiment by using DOX incubated cells were conducted. The impedance of HELF cells remained essentially unchanged with the time in a duration of 20 h after incubation with 0.5 mg/mL of ATF-coupled CSS NPs, which is similar to that of blank HELF in positive control experiment. By contrast, the impedance of HELF cells decreased rapidly with the time when treated with 100 μg/mL of DOX in negative control experiment.

References

1 G. X. Zhao, C. Yuan, C. B. Bian, X. M. Hou, X. L. Shi, X. M. Ye, Z. X. Huang, M. D. Huang, *Protein Expres. Purif.* 2006, **49**, 71.

2 N. J. J. Johnson, A. Korinek, C. H. Dong, F. C. J. M. van Veggel, *J. Am. Chem. Soc.* 2012, **134**, 11068.

3 F. Zhang, R. C. Che, X. M. Li, C. Yao, J. P. Yang, D. K. Shen, P. Hu, W. Li, D. Y. Zhao, *Nano Lett.* 2012, **12**, 2852.

4 N. Bogdan, F. Vetrone, G. A. Ozin, J. A. Capobianco, *Nano Lett.* 2011, **11**, 835.

5 A. G. Dong, X. C. Ye, J. Chen, Y. J. Kang, T. Gordon, J. M. Kikkawa, C. B. Murray, *J. Am. Chem. Soc.* 2011, **133**, 998.

6 X. J. Wang, L. Liu, Y. Luo, H. Y. Zhao, *Langmuir* 2009, **25**, 744.

7 I. Hemmila, S. Dakubu, V. M. Mukkala, H. Siitari, T. Lovgren, *Anal. Biochem.* 1984, **137**, 335.

8 Y. S. Liu, S. Y. Zhou, D. T. Tu, Z. Chen, M. D. Huang, H. M. Zhu, E. Ma, X. Y. Chen, *J. Am. Chem. Soc.* 2012, **134**, 15083.

9 W. Zheng, D. Tu, P. Huang, S. Zhou, Z. Chen, X. Chen, *Chem. Commun.* 2015, **51**, 4129.
